# Supporting Healthier, Sustainable and Equitable Food Environments: A Rapid Review of Australian Local Government Action

**DOI:** 10.3390/nu18162611

**Published:** 2026-08-10

**Authors:** Carmen Vargas, Steven Allender, Miranda R. Blake, Neha Lalchandani, Cindy Needham

**Affiliations:** Deakin Centre for Global Preventive Health and Nutrition, Faculty of Health, Deakin Institute for Health Transformation, School of Health and Social Development, Deakin University, Geelong, VIC 3220, Australia; steven.allender@deakin.edu.au (S.A.); miranda.blake@deakin.edu.au (M.R.B.); n.lalchandani@deakin.edu.au (N.L.); cindy.needham@deakin.edu.au (C.N.)

**Keywords:** local government, food environments, food systems, governance, monitoring

## Abstract

Objective: To synthesise contemporary Australian evidence on how local governments act to support healthier, sustainable and equitable food environments, and to examine the governance arrangements, policy and planning levers, monitoring practices, and implementation conditions shaping local action. Methods: A rapid review of peer-reviewed and grey literature published between January 2015 and February 2026 was performed. Searches were conducted in Medline Complete, CINAHL Complete, Health Policy Reference Centre and Scopus, alongside structured grey literature searches. Included documents addressed Australian LG food environment roles, policies, planning, governance, or monitoring. Findings were narratively synthesised and mapped to national research priorities. Results: Thirty-eight documents were included (23 peer-reviewed studies and 15 grey literature reports). Evidence clustered most strongly around food systems and food security, governance and multisectoral partnerships, and food environment monitoring, particularly food access profiling, affordability monitoring and mapping activities. In contrast, the extent of coverage on statutory planning mechanisms, formal regulatory powers, and consumer behaviour outcomes was limited. Reports of evaluated interventions were concentrated in bounded organisational settings where councils held clearer jurisdiction, such as sport and recreation facilities. Across contexts, LGs were commonly positioned as convenors, brokers and enablers operating within constrained mandates and fragmented multi-level governance settings. Reported enablers included leadership, dedicated resourcing, partnerships and access to local data; common barriers included limited statutory authority, short-term funding and weak vertical policy integration. Conclusions: Australian local governments are active food system actors, but stronger statutory integration, clearer decision pathways, and more consistent monitoring and evaluation are needed to translate local activity into sustained improvements in healthy, sustainable and equitable food environments.

## 1. Introduction

Unhealthy diets, characterized by intakes high in sodium, saturated and trans fats, and added sugars, while low in whole grains, nuts, seeds, fruits, and vegetables [1], contribute significantly to preventable non-communicable diseases (NCDs) such as heart disease, diabetes, and cancer globally [2]. In Australia, the burden attributable to modifiable nutrition-related risks remains high, and excess body weight has recently overtaken tobacco as the leading modifiable risk factor, highlighting the central role of food and nutrition in the nation’s chronic disease profile [3].

A robust evidence base links these burdens to the structure and dynamics of food systems, particularly “food environments” characterized by the availability, pricing, placement, and promotion of foods [4,5]. Foundational conceptual and measurement work defines food (nutrition) environments as the collective physical, economic, policy and sociocultural conditions in which people live and make decisions that influence food choice and nutritional status, and calls for policy and environmental interventions, beyond individual behaviour change, to shift population diets [6]. Subsequent methodological advances have specified measurement domains (e.g., availability, accessibility/affordability, marketing, labelling) and mapped links between outlet density, in-store practices and dietary intake and associated inequities, providing actionable levers for policy change [7,8].

Reflecting this evidence, the global policy agenda has pivoted toward food system transformation. The UN Food Systems Summit (2021) [9] mobilized national dialogues and pathways to deliver healthier, sustainable, and equitable diets, and established follow-up arrangements led by Rome-based UN agencies; the UNFSS+2 Stocktaking Moment (2023) reaffirmed the call to accelerate implementation [10,11]. The Secretary-General’s 2023 report stresses that food system action is a key accelerator for the SDGs and requires multi-level (city–subnational–national) alignment to advance nutrition, equity and sustainability [12].

Australia has begun to institutionalize a focus on ‘systems’ [13] in national prevention agendas. The National Preventive Health Strategy 2021–2030 [14] calls for the adoption of a systems-based approach that addresses social and environmental determinants (including food) and sets targets to halt and reverse obesity trends through cross-sector action. A national preventive-health monitoring dashboard now tracks progress toward these targets, reinforcing the need to translate strategy into local government (LG) planning, procurement and regulatory practice, where many food environment levers sit [15]. In parallel, international networks such as INFORMAS have developed benchmarking frameworks (e.g., the Government Healthy Food Environment Policy Index: Food-EPI) that assess and prioritize government policies across domains such as composition, labelling, marketing, retail, pricing, and public-sector food provision, approaches already applied in Australia and comparable settings [16,17,18].

Recent national engagement with Australian LGs and researchers has identified a shared set of research priorities to support the development of healthy, sustainable and equitable food environments. However, evidence relevant to LG-level food environment action remains fragmented across peer-reviewed studies, government strategies, program evaluations and practice-based documentation, limiting its accessibility and utility for LG decision-makers. This fragmentation constrains governments’ ability to identify documented approaches, benchmark progress, prioritize investment, and consider potential implications for scaling, which are challenges that international monitoring initiatives seek to address through standardized assessment and reporting. Previous reviews in Australia have examined local government food policies and interventions, highlighting important progress and challenges [19,20,21]. This rapid review builds on that foundation by incorporating the latest evidence through to early 2026, reflecting post-pandemic and UN Food Systems Summit developments; integrating practice-based grey literature often omitted from academic syntheses; and organizing all evidence around nine nationally endorsed research priorities developed by Australian local government and research stakeholders in 2025. It is therefore designed to provide a timely, policy-ready synthesis of contemporary Australian evidence. Conceptually this review is grounded in a multi-level food systems perspective and informed by established food environment frameworks that emphasize the interaction between physical, economic, policy, and sociocultural determinants of diet [22]. Analytically, it is guided by nationally identified LG research priority themes [23], which we use to organize and interpret the evidence (Table 1). These themes align with established food environment models and with recognized LG governance roles, providing a lens through which to examine not only what local actions are being taken, but also the conditions that shape their implementation [24]. By applying this framework across peer-reviewed and grey literature, the review documents who is doing what, where, and with what reported outcomes across Australian LGs. In doing so, it links national prevention commitments to local food environment levers, identifies reported implementation enablers and barriers across diverse contexts, and highlights evidence and policy gaps to inform monitoring, implementation and future investment.

This rapid review responds to an urgent need for compiled evidence to inform local government planning cycles and national policy initiatives (e.g., National Preventive Health Strategy 2021–2030) in a timely manner. Specifically, it addresses the following research questions:

- How is evidence of Australian local government action distributed across nationally identified priority themes for healthier, sustainable and equitable food environments?
- What governance arrangements, statutory and non-statutory planning levers, and/or intervention settings are reported across Australian local governments seeking to influence food environments?
- What implementation enablers, barriers, and evidence gaps are reported across the contemporary evidence on Australian local government food environment initiatives?

## 2. Materials and Methods

### 2.1. Study Design

A rapid review with an evidence-mapping and narrative-synthesis approach was conducted to characterise the distribution, scope and reported features of contemporary Australian evidence relevant to local government action supporting healthier, sustainable and equitable food environments. The review was designed to map available evidence and implementation experience rather than determine intervention effectiveness or evidence certainty. Rapid reviews use systematically organised but pragmatically streamlined methods to provide timely, decision-ready evidence for policy and practice, particularly when traditional systematic reviews are infeasible due to time or resource constraints [20,21]. This approach is widely endorsed for public health and policy-focused questions requiring a balance between methodological rigour and timely knowledge translation [22]. The review was conducted in accordance with methodological guidance from the Cochrane Rapid Reviews Methods Group [23] and informed by the Preferred Reporting Items of Systematic Reviews and Meta-Analyses (PRISMA) guidelines [24] with tailored adaptations appropriate to a rapid review design.

### 2.2. Review Scope and Research Framework

The review focused exclusively on Australian-based evidence relevant to LG policy, programs, planning responsibilities and governance levers. The scope of the review was defined a priori and anchored to the Local Governments’ National Research Priorities for supporting healthier, sustainable and equitable food environments. These priorities were not developed by the review team for this study. Rather, the nine priority themes were generated through a deliberative, stakeholder-informed process at the first national Local Governments Creating Healthier Food Environments Symposium [25], held in Melbourne and online in February 2025. The symposium brought together local government practitioners, public health researchers and policymakers, who participated in structured workshops and consensus voting to identify shared research priorities for local government action and evidence generation [19]. Participants engaged in a structured workshop to identify current evidence needs, policy and practice challenges, and priority areas for future research. Workshop outputs were synthesised and organised into preliminary themes, which were then circulated via survey to all registered Symposium participants to assess consensus. Consensus was reached, resulting in nine agreed priority themes. Theme definitions were subsequently developed to clarify the scope of each priority area and support consistent interpretation and application within this review.

The research priority definitions were considered appropriate as an a priori analytical framework because they were developed through a documented, stakeholder-informed national process and directly reflected the policy and practice context being mapped. The nine priorities were therefore used as an organisational lens to categorise the scope and distribution of available literature (Table 1), not as a quality appraisal tool, evidence-grading framework, or basis for assessing intervention effectiveness.

### 2.3. Eligibility Criteria

The eligibility criteria were based on the Population/problem, Interest, Context and Scope and Time (PICoST) approach [26]. The PICoST framework is particularly well suited to complex, policy and systems-focused public health questions, where interventions are heterogeneous, non-experimental, and embedded within organisational or governance contexts rather than controlled clinical settings [27]. Table 2 presents the inclusion and exclusion criteria.

### 2.4. Search Strategy

We systematically searched Medline Complete, CINAHL Complete, Health Policy Reference Centre (EBSCOhost), and Scopus for peer-reviewed literature published between January 2015 and 27 February 2026 that examined Australian food environment research, policies, programs or interventions relevant to LG action. The search timeframe was selected to capture contemporary evidence aligned with recent developments in food environment policy, planning and governance in Australia.

The search strategy was informed by previous systematic and rapid reviews examining food environments, LG policy levers and population-level nutrition interventions [28,29]. Search terms combined controlled vocabulary and free-text keywords relating to LG, food environments, food systems, policy, planning, governance and Australia (Supplementary Table S1). One reviewer (CV) independently developed and ran the search strategy, with support from an experienced academic librarian to refine, calibrate and adapt search terms across databases.

Consistent with best practice for rapid reviews in applied policy contexts, a structured grey literature search was developed to minimise publication bias and capture practice-based evidence that is often absent from academic databases [22,30]. The grey literature search plan was developed to incorporate two different search strategies (Supplementary Table S2): (1) grey literature databases (Trove and Australian Policy Online [APO]) and (2) a customised Google search. All grey literature search queries, including Google-based queries, were executed in private browsing mode to reduce personalization. For Google-based queries, we pre-specified review of the first 10 results pages, equivalent to 100 hits per search, to enhance repeatability and manage workload. The exact search terms and approach are detailed in Supplementary Table S2.

### 2.5. Screening and Study Selection

We applied two independent screening workflows: (1) peer-reviewed academic literature and (2) a parallel workflow for grey literature. Each workflow followed a two-stage screening process, adapted to the characteristics of the respective evidence source.

Database searches were imported to Covidence [31], where duplicates were removed, and screening was conducted. We followed a rapid review hybrid screening model [23]: two reviewers (CV, CN) independently screened all titles and abstracts for eligibility. At full-text screening, one reviewer (CV) screened all documents; additionally, 50% of full texts (randomly selected) were independently screened by a second reviewer (CN) to enhance rigour. Disagreements at any stage were resolved through discussion or with a third reviewer (SA) as needed. Grey literature items rarely have structured abstracts; therefore, we screened the title/landing page and the first available synopsis (executive summary, overview page, or table of contents). One reviewer (CV) screened all potential inclusions; a second reviewer (CN) verified all full-text exclusions and applied coded reasons (e.g., non-food environment scope; undated/obsolete).

### 2.6. Data Extraction

A data extraction template was developed to capture information relevant to LG food environment action. In line with rapid review methods [23], one author (CV) extracted data from all sources into an Excel spreadsheet, with a 20% random sample of extractions independently verified by a second reviewer (CN) to balance rigour and timeliness in the rapid synthesis.

For peer-reviewed literature, extracted data included publication characteristics (type and year); study or document aims and design; use of conceptual or theoretical frameworks; LG roles, responsibilities and policy or planning levers addressed; details of the intervention, policy or practice (including name and key characteristics); funding sources and partner organisations; reported outcomes and indicators described by individual studies as effectiveness-related; geographic and sociodemographic context; and reported implementation enablers, barriers and considerations.

For grey literature, the extraction template was adapted to reflect the practice-oriented purpose of these sources. Extracted data included document type and intended use (e.g., situational analysis, planning guidance, program report or monitoring output); geographic scope and target populations; food system and food environment domains addressed; LG roles, governance arrangements and multisectoral partnerships described; data sources used (e.g., audits, mapping exercises, administrative data, community consultations); actions, strategies or initiatives reported; proposed or applied monitoring indicators; implementation considerations; and evidence of evaluation or review where available. Traditional study design and effectiveness fields were considered inappropriate, recognising grey literature as a form of practice-based evidence designed to inform planning, decision-making and implementation rather than to test intervention effects.

### 2.7. Evidence Synthesis

A narrative synthesis approach was used to integrate findings across both peer-reviewed and grey literature, supported by thematic mapping to the Local Governments’ National Research Priority Themes (Table 1). Narrative synthesis is widely recommended for policy-oriented reviews where methodological diversity and contextual variability preclude meta-analysis [30]. Extracted data were coded and summarised in tables and matrices to address the review aim and research questions by: (1) mapping the content of included policies, programs and practice documents to the nationally identified priority themes, based on the extent to which they addressed each theme and the level of substantive detail provided (i.e., not addressed, primary, secondary); (2) synthesising the reported governance arrangements, planning levers, evaluated activity and documented strategies across Australian LGs; and (3) identifying contextual enablers, barriers and evidence gaps, including priorities for future research, monitoring and practice development.

Documents were coded against each of the nine priority themes as having primary, secondary or no substantive relevance according to predefined coding criteria. Primary relevance indicated that the theme was an explicit or central focus and included substantive detail, such as actions, implementation, evaluation or monitoring. Secondary relevance indicated that the theme was addressed indirectly or tangentially, including brief references or content embedded within broader wellbeing, sustainability or food systems strategies. Themes were coded as not addressed where no meaningful content mapped to the priority theme. Coding was undertaken by one reviewer (CV), who applied the coding guide (Table S5) to each document through full-text review, assigning a code for each theme and recording evidence notes where relevant to support coding decisions. A sample of coded documents was reviewed by a second reviewer (CN) to assess consistency in code application and interpretation. Any discrepancies were discussed and resolved by consensus. No formal critical appraisal or risk-of-bias assessment was undertaken. Documents were therefore not weighted by methodological quality, and the synthesis describes the volume, distribution and reported characteristics of the available evidence rather than its certainty or comparative strength.

## 3. Results

**Figure 1:**
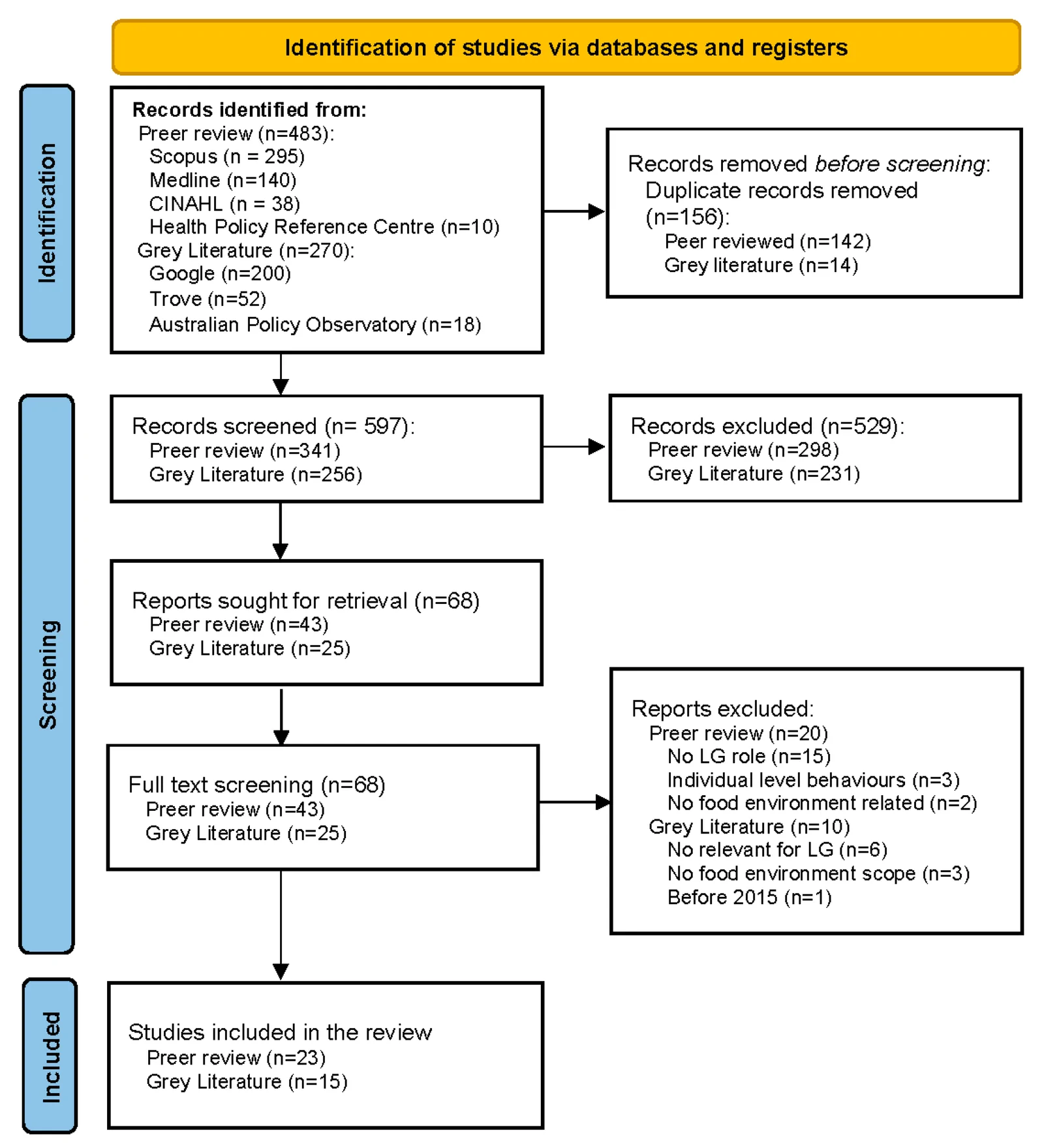
PRISMA diagram of the systematic review process for this review.

A total of 597 documents were considered for screening after duplicates were removed. Of these, 341 peer-reviewed titles and abstracts along with 257 grey literature reports were screened, and 43 peer-reviewed and 25 grey literature reports were fully assessed against the eligibility criteria (Table 2). Finally, 23 peer-reviewed articles and 15 grey literature reports were included in our rapid review. A PRISMA flow diagram (Figure 1) illustrates the search results and data collection process.

### 3.1. Characteristics of Included Documents

The included documents comprised a heterogeneous but conceptually coherent body of evidence spanning peer-reviewed studies (n = 23) and grey literature (n = 15) produced between 2016 and 2025. Peer-reviewed articles most frequently examined governance and partnership arrangements (n = 9), policy mapping or policy analysis (n = 8), qualitative case studies (n = 7), and intervention implementation or outcomes (n = 4). Peer-reviewed categories were descriptive and not mutually exclusive; therefore, totals do not sum to 23. The grey literature documents comprised statutory policy documents (n = 2), regional strategies (n = 2), monitoring reports (n = 8), planning tools (n = 1), and practice-based grey literature (n = 2). Collectively, the documents capture how Australian LGs engage with healthy food environments, food security, and broader food systems through statutory planning, service provision, partnerships, advocacy, and facilitation roles. The peer-reviewed literature was dominated by Victorian studies (n = 13), with a smaller but more geographically mixed set from New South Wales (n = 2), Western Australia (n = 2), the Northern Territory (n = 1), and cross-jurisdictional studies spanning the Australian Capital Territory and New South Wales (n = 1), New South Wales and Queensland (n = 1), and New South Wales and Victoria (n = 3). Notably, no included studies used randomized controlled trial designs, and prospective or longitudinal evaluations were uncommon, indicating that the peer-reviewed evidence base was primarily descriptive, policy-oriented, or implementation-focused rather than designed to test causal effects. In contrast, the grey literature was concentrated largely in Tasmania (n = 9), with fewer documents from Victoria (n = 4) and national-level sources (n = 2). Eight included community food access profiles were produced through the Healthy Food Access Tasmania initiative and used a shared program and reporting approach. They were retained as separate documents because they described distinct local government contexts, but they should not be interpreted as independent evaluations.

Across both evidence streams, LGs were commonly positioned as enablers and convenors, operating within constrained mandates and fragmented governance settings. Peer reviewed studies (Table 3 and Supplementary Table S3) primarily focused on governance analysis (n = 9) [32,33,34,35,36,37,38,39,40], policy mapping (n = 8) [38,41,42,43,44,45,46,47], or qualitative case study designs (n = 7) [33,39,40,45,48,49,50], with relatively few studies evaluating intervention implementation or outcomes (n = 4) [51,52,53,54]. Grey literature (Table 4 and Supplementary Table S4) contributed comparatively richer detail on local context realities (n = 5) [55,56,57,58,59,60,61,62,63,64,65,66,67,68,69] and monitoring practices, particularly in regional, rural, and remote settings (n = 12) [55,56,59,61,62,63,64,65,66,67,68,69]. The diversity of included documents reflects both the complexity of food systems action and the institutional constraints under which LGs operate.

### 3.2. Mapping Content to Nationally Identified Priority Themes

**Figure 2:**
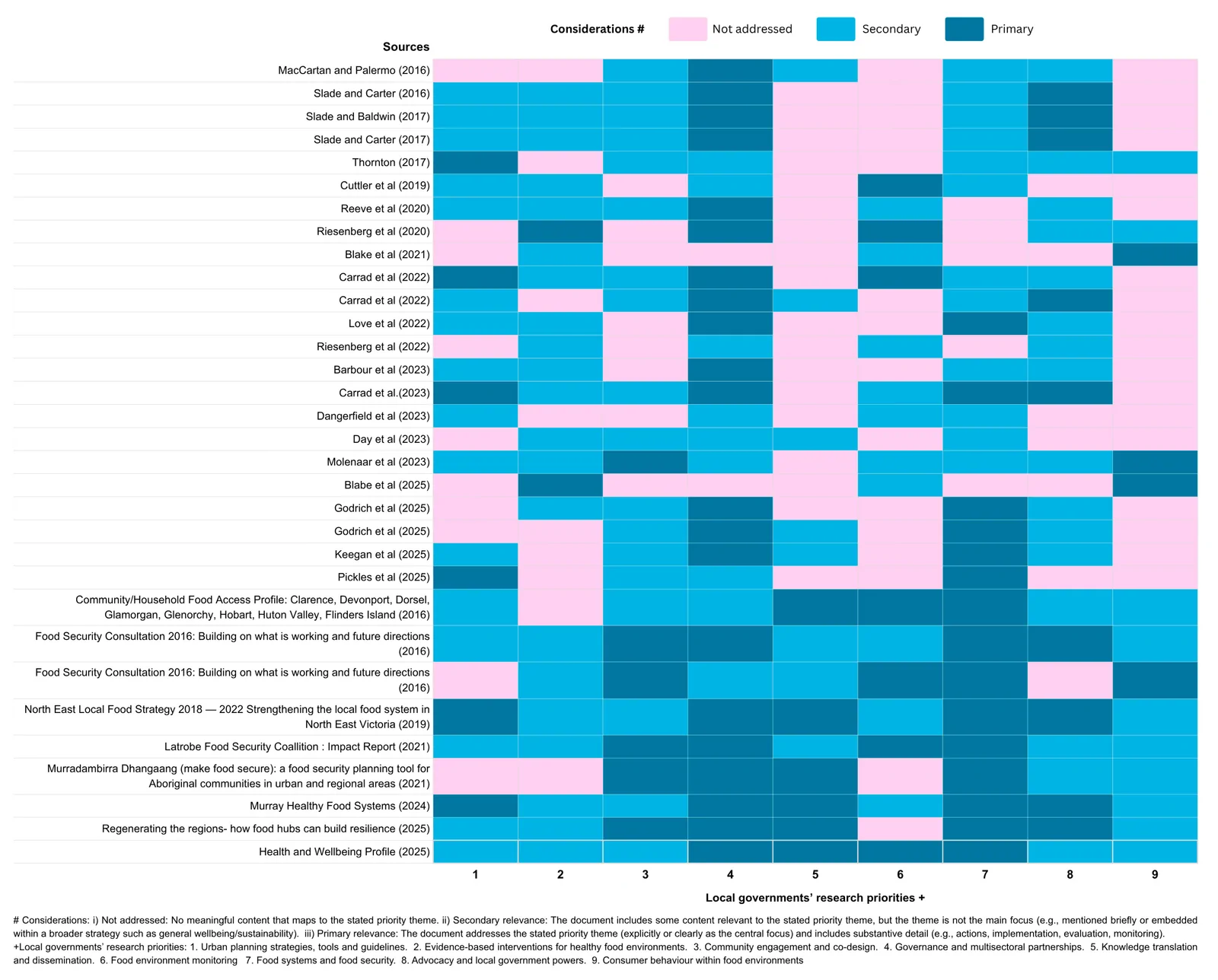
Mapping of included studies and reports to local governments’ healthy food environment research priorities [32,33,34,35,36,37,38,39,40,41,42,43,44,45,46,47,48,49,50,51,52,53,54,55,56,57,58,59,60,61,62,63,64,65,66,67,68,69].

When mapped against nationally articulated priority themes for LG action on healthy food environments and food systems, content clustered unevenly across themes (Figure 2). Documents were classified as having primary, secondary, or no relevance based on the extent to which they addressed each theme and the level of substantive detail provided (Table S6).

The greatest volume of mapped content related to food systems and food security, governance and multisectoral partnerships, and food environment monitoring. Numerous documents reported LG engagement in food access profiling, affordability monitoring, and descriptive mapping of retail and supply conditions, particularly through initiatives such as the Healthy Food Access Tasmania profiles [57,58,66,67,68,69,70,71] and regional food basket studies [49]. These documented approaches were closely aligned with national calls to strengthen sub-national food systems monitoring but were not consistently embedded within statutory planning instruments. Governance and partnership themes were similarly prominent. Peer-reviewed studies frequently reported coalitions, inter-governmental collaborations, and place-based partnerships as mechanisms through which LGs influence food systems [37,38,40]. Grey literature reinforced this pattern, documenting collective impact models [63], Aboriginal-led governance approaches [62], and cross-sector reference groups as reported enabling structures for food security action [62,63,65].

By contrast, urban planning strategies, advocacy using formal statutory powers, and consumer behaviour change within food environments were addressed more selectively. Although planning was frequently acknowledged as an important lever, detailed analysis of zoning, development controls, and land use planning remained rare and often constrained by state-level legislation [41,42,50]. Behavioural dimensions were typically inferred through participation or sales data rather than examined as primary outcomes [51,52,53,54].

### 3.3. Governance Arrangements, Planning Levers, Interventions, and Strategies

Synthesis across the included documents suggests that Australian LG action on food environments is shaped less by discrete interventions than by institutional positioning within multi-level governance systems. Governance and partnership activity was frequently reported in contexts where LGs acted as brokers and convenors, leveraging relationships across health services, community organisations, producers, and state agencies, rather than acting independently [33,43,44].

Planning and regulatory levers were unevenly operationalised. While statutory instruments such as Municipal Public Health and Wellbeing Plans provided an entry point for action [44,46], LG influence over the commercial retail environment was frequently described as indirect and constrained, for example through planning approvals for new supermarkets or fresh food outlets in underserved areas [36,38]. Consequently, many strategies were concentrated on organisational food environments, council-owned assets, procurement practices, and facilitation of community initiatives, rather than upstream structural change [52,53].

Evaluated activity was concentrated in discrete, bounded settings, particularly healthy food and drink initiatives in sport and recreation facilities, where local governments held relatively clear jurisdiction and implementation could be phased and monitored [53,55]. In contrast, broader food system strategies and food security initiatives relied more on systems approaches such as coordination, capacity building, and enabling conditions to make action possible. Reported outcomes were commonly framed as institutional or governance change rather than community-level health outcomes [37,64].

### 3.4. Contextual Enablers, Barriers, and Evidence Gaps

Across the peer-reviewed and grey literature, key enabling factors included political and organizational leadership, dedicated funding and staffing, strong partnerships, access to local data, and, particularly in regional and remote areas, place-based knowledge [26,33,36]. In regional and remote contexts, place-based knowledge, strong community networks, and adaptation to local supply conditions were particularly important [38,53,54,62,63,64,65,66,67]. Conversely, barriers were predominantly structural, including limited statutory authority, short-term funding, workforce turnover, fragmented policy frameworks, and weak vertical coordination across government levels [34,36,42]. These constraints often pushed LGs toward smaller, feasible local actions rather than transformative system-level interventions for structural reform.

Significant evidence gaps were identified. There remains a lack of longitudinal evaluation, limited use of environment-level indicators, and insufficient integration of equity-focused outcomes beyond descriptive framing [41,42]. This gap partly reflected the limited use of prospective, controlled, or quasi-experimental designs, with most studies relying on cross-sectional, qualitative, policy mapping, or descriptive monitoring approaches. While equity was frequently invoked, particularly in food security and Aboriginal-led initiatives, few studies examined how equity goals translated into sustained structural change [49,60].

Priorities for future research and practice development include: (i) stronger integration of food systems objectives into statutory planning; (ii) development of standardized, LG-appropriate monitoring frameworks; (iii) evaluation of governance and systems-change mechanisms (not only interventions); and (iv) long-term sustained investment, capacity building, co-design, and Indigenous-led governance models aligned with self-determination and place-based approaches.

## 4. Discussion

This review examined how Australian LGs engage with healthy food environments and food systems, and how existing evidence aligns with nationally articulated priority themes. It provides a timely synthesis of contemporary Australian evidence (2015–2026) on LG action to promote healthier, sustainable, and equitable food environments particularly given the increasing recognition of health and wellbeing considerations within planning systems and policy frameworks across multiple Australian states, including Victoria, Queensland and New South Wales [72,73,74]. Building on previous syntheses, including policy mapping [20] and governance analyses [21], this review brings together peer-reviewed and grey literature and organises the evidence according to contemporary national priorities, offering a practice-oriented framework for interpreting LG action. The findings indicate that the mapped literature was concentrated in relation to food environment monitoring, multisectoral partnerships, food security activity, and organisational settings, and that the extent of coverage remains limited for statutory planning mechanisms, equity outcomes, and longitudinal impact. They also highlight governance arrangements, including brokering, convening and vertical integration across government levels, as reported implementation conditions shaping what LGs can implement and sustain. In this way, the review shifts attention from individual programs alone to the broader system conditions shaping local action, while providing a synthesis that can inform policy, planning and future research.

Overall, the evidence base suggests that LGs’ influence over healthy food environments is mediated less by direct regulatory power than by institutional position within broader governance systems. Evidence is concentrated in areas where councils can act through coordination, data generation, and organisational control, whereas statutory planning levers and consumer-facing outcomes remain less examined empirically. Notably, knowledge translation and dissemination, and consumer behaviour within food environments were seldom the focus of peer-reviewed research. This likely reflects the academic literature’s emphasis on policy and planning processes, while knowledge translation activities often occur within practice or intermediary organisations and may be more visible in grey literature than in academic publications. Similarly, the limited measurement of dietary, obesity-related or other population-level outcomes may explain the paucity of studies examining consumer behaviour change resulting from LG food environment initiatives. Although locally responsive monitoring and place-based initiatives are frequently reported, particularly outside metropolitan areas, their translation into sustained action is often constrained by short-term funding, limited statutory authority, and weak integration across policy levels. The clustering of evaluated activity in council-owned or organisational settings further underscores how authority, jurisdiction, and governance alignment shape what is practically achievable. This pattern reinforces that the current evidence base is predominantly descriptive, with limited evaluation of population-level impacts. The absence of outcome-focused evidence constrains the ability to assess reported outcomes of local government actions beyond organisational or process-level change. Viewed collectively, these patterns point to enduring gaps in longitudinal evaluation, environment-level indicators, and the operationalisation of equity goals, highlighting the need to move beyond articulated intent towards governance-embedded mechanisms that support sustained and measurable change. Our findings reinforce that local government actions can be interpreted through a socio-ecological and multi-level governance lens [5,75], wherein underlying structural factors (e.g., legislative constraints, resource dependencies, and partner networks) shape what actions are feasible. This theoretical framing highlights that observed local actions, often facilitative or coordinative, reflect broader institutional determinants despite an overall policy ambition to create healthier, sustainable, and equitable food environments.

The evidence base in this review reflects a pattern of LGs acting primarily through facilitative and coordinative roles within complex and constrained governance settings, rather than through direct regulatory control. The literature was most concentrated around food systems and food security, governance and multisectoral partnerships, and food environment monitoring, while comparatively less empirical attention has been given to statutory planning levers and consumer behaviour outcomes. Monitoring activities are widespread and locally responsive, particularly in regional and rural contexts, yet are rarely institutionalised within statutory planning frameworks or linked to formal decision-making thresholds. Where evaluated activity exists, it is concentrated in settings under clear LG jurisdiction, highlighting the importance of authority and control for translating policy intent into measurable change. Across the literature, enabling conditions such as leadership, sustained resourcing, robust partnerships, and access to local data consistently shaped the scope and durability of reported activity, while structural constraints curtail potential implications for scale and sustainability, including limited statutory authority, short funding cycles, workforce instability, and weak vertical policy integration. Persistent gaps in longitudinal evaluation, environment-level indicators, and the measurement of equity outcomes suggest that while equity and systems change are frequently articulated as goals, they remain insufficiently operationalised within LG food policy and practice.

Our findings show that the scope and depth of local action are less a function of policy ambition than of institutional authority and regulatory reach. These results are consistent with prior research [70,71], reinforcing that LG action on healthy food environments is shaped by the interaction between available levers, such as procurement, council-owned facilities, and discretionary programs, and structural constraints, including planning legislation and limited authority over the commercial retail environment. Previous studies have highlighted that while LGs can act decisively within domains under their direct control, their ability to influence broader food retail landscapes is mediated by state planning frameworks and market governance arrangements [36,41,46].

International and Australian studies have consistently argued that while LGs play a critical role in generating context-specific data and intelligence, particularly through food access and affordability monitoring, such evidence has limited impact unless it is embedded within statutory planning instruments and linked to decision-making processes [38,46,72]. Our review extends this insight by showing that although monitoring practices are widespread and increasingly sophisticated, particularly in regional contexts, they are rarely connected to explicit policy triggers or accountability mechanisms. This finding reinforces food environment and systems research by emphasising that local monitoring, while necessary, is insufficient to drive upstream change unless it is embedded within coordinated governance arrangements and statutory planning cycles operating across multiple levels of government.

The concentration of evaluated activity in bounded settings, such as council-owned sport and recreation facilities, mirrors patterns observed in the broader implementation and public health intervention literature [73]. Previous research has shown that interventions are more likely to be evaluated where settings, populations, and lines of responsibility are clearly defined, implementation is standardised, and causal pathways are more easily specified [53,55]. The limited evaluative evidence for more systemic or place-wide food security strategies identified in this review reflects these methodological and institutional challenges, rather than an absence of action. The absence of randomised trials and the limited number of prospective or quasi-experimental evaluations are unsurprising given the complexity of local government food environment action, but they limit causal inference about the reported outcomes of specific policies or governance approaches.

The prominence of partnerships and coalition-based models across the reviewed literature aligns with governance scholarship that characterises LG as a convenor and broker within place-based systems, rather than a sole actor driving change through formal regulation [74]. Our findings corroborate this framing, showing that collaboration and coalition-building function as core modes of LG practice in food systems governance. Prior work highlights the importance of multisectoral partnerships in addressing complex food security challenges, particularly in regional, rural, and remote contexts with limited statutory leverage [19,38,40]. Current evaluations of multisector networks show that collective initiatives can support coordination, knowledge exchange, and shared accountability even in the absence of strong regulatory authority [74]. Together, this body of evidence suggests that partnerships are not simply compensatory mechanisms for weak levers, but a documented governance strategy for addressing the complexity of food systems challenges.

This review extends the literature by demonstrating the extent to which practical, operational knowledge about LG action resides in grey literature, and by showing how this practice-based evidence complements, and in some cases exceeds, peer-reviewed reporting in contextual richness and implementation specificity. While previous reviews have acknowledged the value of grey literature for capturing real-world policy activity [75,76], our synthesis makes its contribution more explicit by highlighting the unique insights grey sources provide into local decision-making processes, resourcing arrangements, and monitoring practices that are frequently under-reported in academic studies. These findings underscore the importance of deliberately integrating grey and peer-reviewed evidence when assessing LG responses to complex, systems-level public health challenges.

### 4.1. Implications for Policy and Practice

The findings of this review have several potential implications for policy and practice. There is a clear need to strengthen the statutory and strategic integration of food systems and food environment objectives within core LG instruments, where feasible, with explicit accountability and review cycles. As an example of statutory integration, one Victorian LG incorporated healthy food access objectives into its Municipal Public Health and Wellbeing Plan, ensuring that food environment considerations were included in formal planning and evaluation cycles [60]. Moving beyond data collection alone, monitoring outputs, such as food access and affordability profiles or spatial mapping, should be systematically linked to decision points, including procurement standards, council-owned facility policies, funding criteria, and planning advocacy priorities. For instance, one regional council used annual food access profile data mapping healthy and unhealthy retail outlets to advocate for changes in local zoning guidelines and budget allocations to attract healthier food retail options to underserved areas [49,57,58,66,67,68,69,70,71].

Given ongoing constraints on regulatory authority, councils are likely to achieve greatest impact by scaling action in domains where they hold clearer control, such as procurement, events, facilities, and community infrastructure, while simultaneously pursuing coordinated advocacy for state-level reforms in land use planning and retail regulation. The review also underscores the importance of investing in governance capacity, including dedicated coordination roles, formalised partnership structures, and mechanisms that support vertical integration across local, state, and federal priorities. Long-term funding and institutional continuity are also critical, as short-term project-based approaches may undermine sustained systems change, highlighting the need for stable investment models that support enduring partnerships, workforce retention, and iterative policy development.

Finally, equity and self-determination must be embedded more deliberately in policy and practice through co-design with priority populations, Aboriginal-led governance approaches, measurable indicators, sustained resourcing, and long-term commitment. For example, the Murradambirra Dhangaang (‘make food secure’) planning tool [60], developed through an Aboriginal-led process, provides a model for incorporating Indigenous leadership and cultural context into local food security planning. LGs can support such frameworks by partnering with Aboriginal Community-Controlled Organisations and embedding self-determination principles in their food system strategies. More broadly, equity objectives should be more than aspirational; LGs can operationalise equity by incorporating explicit equity indicators into monitoring frameworks, such as measures of healthy food access gaps for low income, rural and remote, Aboriginal and Torres Strait Islander, or other priority communities, alongside targets and accountability mechanisms to reduce disparities in food environments. For example, local plans could track improvements in healthy food availability or affordability for priority populations, thereby embedding equity more directly into decision making.

### 4.2. Strengths and Limitations

Our rapid review provides a timely synthesis of contemporary Australian evidence. Including both peer-reviewed and grey literature strengthened the practice relevance of the findings for LG decision-making, and mapping evidence to nationally agreed priority themes provided a clear organizing framework. These types of reviews are essential to support governments to meet the requirements of systems change reflected in national prevention and obesity strategies [77]. A key limitation is that the evidence base was heterogeneous, limiting direct comparison across outcomes, and many sources reported activities and outputs rather than measured impacts. We did not conduct a formal quality or risk-of-bias assessment, reflecting the rapid review design and the methodological heterogeneity of included peer-reviewed and grey literature. Although second-reviewer checking of a sample of coded documents strengthened consistency, the primary reliance on one reviewer for coding may have introduced some interpretive bias in how documents were classified across priority themes. Consequently, this review cannot determine the certainty, comparative strength or effectiveness of the available evidence; findings should therefore be interpreted as a synthesis of the distribution and characteristics of the literature rather than as an assessment of intervention effectiveness. Additionally, the methodological transparency of grey literature varied, and the rapid review approach may have missed some materials, particularly web-only content, meaning the review may under-represent unpublished local initiatives. Finally, although we took steps to improve the transparency and repeatability of the grey literature search, reliance on Google-based searching introduces some uncertainty because search results are shaped by dynamic algorithms and may vary over time. To mitigate this limitation, we documented the search terms, sources and screening approach in detail in Supplementary Table S2.

The evidence base also showed a geographic skew, with most peer-reviewed studies from Victoria and many grey literature reports from Tasmania, which may limit the generalizability of findings to under-represented Australian states and territories. The thematic distribution identified in this review may partly reflect jurisdiction-specific research programs and monitoring initiatives rather than the national distribution of local government activity. Because LG structures, statutory responsibilities and policy contexts vary by jurisdiction, the findings should be interpreted with this context in mind. There was also a temporal skew within the grey literature, with over half of the included grey literature documents published in 2015–2016 as part of the Tasmanian community food access profiles. These sources provide valuable contextual insights but may not fully reflect post-2020 policy, planning or food system developments. We therefore use “contemporary” to denote evidence published within the 2015–2026 review window, while acknowledging that some included sources are older and context-specific.

### 4.3. Future Research

Future research should strengthen evaluation of LG food environment action by linking policies, programs and governance approaches to measurable outcomes, including dietary behaviours, obesity-related indicators, environmental change, equity and sustainability. Given the complexity of local policy and systems interventions, RCTs may not always be feasible or appropriate; however, greater use of prospective, natural experiment, quasi-experimental, realist and mixed-method designs would improve causal inference and understanding of how change occurs. These approaches should be supported by standardised, LG-appropriate monitoring indicators that can be embedded in routine policy, planning and program evaluation, enabling councils to track progress over time and use evidence more directly in decision-making. The synthesis presented here can help inform emerging national monitoring infrastructure, such as the Australian Food Atlas, by identifying LG-relevant indicators, evidence gaps and priority domains for routine food environment monitoring.

Future studies should also examine the governance and implementation conditions that enable sustained systems change, including resourcing, policy alignment, statutory authority, partnership models and vertical coordination across government levels. In particular, stronger evidence is needed on how statutory planning mechanisms, including zoning and development controls, can be applied and evaluated to influence retail food environments. Evaluation should move beyond program outputs to assess environmental changes, such as availability, affordability and marketing exposure, as well as distributional impacts for priority populations. Further research should also document and evaluate Aboriginal-led and community co-design approaches, including governance models that support self-determination and place-based implementation. Finally, given the geographic concentration of current evidence in Victoria and Tasmania, studies in under-represented jurisdictions, such as Queensland, South Australia and Western Australia, are needed to strengthen generalisability and clarify how different state and territory policy contexts shape LG food environment action.

## 5. Conclusions

Australian LGs are actively engaged in food systems and food security action, particularly through monitoring, partnerships, and initiatives within organizational settings, but these efforts remain unevenly embedded in statutory planning and are constrained by multi-level governance settings. Overall, the findings imply that improving food environments through LG action is contingent on strengthening policy integration and governance structures, with sustained attention to health, sustainability, and equity outcomes. Clear decision pathways linked to monitoring are likely to be critical for translating current activity into sustained, measurable improvements in healthy, sustainable and equitable food environments. A more consistent evaluation and monitoring agenda, aligned to nationally identified priorities, would further support benchmarking, knowledge translation, and more rigorous evaluation of promising local government approaches before wider implementation or scale-up.

## Figures and Tables

**Table 1:** Local governments’ research priorities for establishing and supporting healthier, sustainable and equitable food environments [19].

Principles	Outline
Urban planning strategies, tools and guidelines	Ways LGs use land use planning, zoning rules, development controls, and design standards to shape where food outlets are located, how people access them, and how the built environment supports healthy and sustainable food choices
Evidence-based interventions for healthy food environments	Policies, programs and actions that are designed and implemented using the best available research and local data, and that have been shown to improve access to healthy foods, reduce exposure to unhealthy foods, and promote healthier dietary behaviours
Community engagement and co-design	Processes where local communities, including priority and marginalized groups, are actively involved in identifying issues, generating ideas, and jointly designing food-related policies, spaces and programs so that solutions reflect local needs, values and lived experience
Governance and multisectoral partnerships	The structures, processes and collaborations through which LG works with other sectors (e.g., health, education, transport, business, housing, environment, community organizations) to plan, implement and coordinate actions that influence the food environment
Knowledge translation and dissemination	Activities that turn research findings, monitoring data and practice insights into clear, usable information, tools and guidance that can be shared with decision-makers, practitioners and communities to support better food-related policies and practices
Food environment monitoring	The ongoing collection, analysis and reporting of data on aspects of the local food environment (such as outlet types, locations, marketing, pricing and availability of healthy options) to track changes over time and assess the impact of policies and interventions.
Food systems and food security	Understanding and acting on the full system that produces, processes, distributes, sells and disposes of food, with a focus on ensuring that all people have reliable, dignified access to sufficient, safe, nutritious and culturally appropriate food, now and in the future
Advocacy and LG powers	LGs use their legal powers, influence and leadership, such as planning controls, licensing, procurement, and public communication, to promote healthier and more sustainable food environments, and to advocate for supportive policies at state and federal levels
Consumer behaviour within food environments	How people make choices about what, where and how they eat in response to the food options, prices, marketing, cultural norms and physical design of their surroundings, and how LG actions can shape these behaviours towards healthier and more sustainable patterns.

**Table 2:** Inclusion and exclusion criteria for the rapid review using the PICoST framework.

	Inclusion	Exclusion
Problem	Focused on Australian LGs, LG authorities, councils, or LG-led or LG-involved initiatives Addressed food environments or food system issues relevant to populations influenced by LG action (e.g., communities, priority populations, rural or socioeconomically disadvantaged groups)	Focused solely on community-level dietary behaviours or nutrition outcomes without reference to environmental, policy, or systems-level determinants Addressed food or nutrition issues with no relevance to LG roles or levers
Interest	Examined LG policies, strategies, programs, planning tools, governance mechanisms, interventions, advocacy actions, or monitoring activities aligned with at least one national research priority theme (Table 1)	Articles without empirical evidence, policy analysis, or documented programs Publications addressing food production, agriculture, or sustainability without explicit linkage to food environments or LG action
Context	Conducted within an Australian context, including urban, regional, rural, or remote settings Relevant to LG statutory responsibilities, decision-making, or operational practice	Studies conducted entirely outside Australia Analyses focused exclusively on federal or state policy with no implications for LG practice
Scope	Peer-reviewed research (qualitative, quantitative, mixed-methods, or review articles). Grey literature, published by a government or non-government organisation (NGO) at either the federal, state or LG level within Australia including government reports, LG plans and strategies, statutory authority publications, and NGO reports Case studies or evaluations documenting LG–related food environment initiatives.	Conference abstracts, books, book chapters, commentaries, opinion pieces, letters, editorials, protocols, theses, modelling, laboratory-based studies, and reviews Unverifiable documents (year lacking) or sources without sufficient methodological, policy, or program detail Grey literature only: web-only items without a file (i.e., PDF, Word or equivalent)
Time	Evidence published between January 2015 and 27 February 2026	Evidence published prior to 2015

**Table 3:** Outline of included peer-reviewed articles (n = 23).

Authors (Year)	Aim	Study Design (State/Territory Analysed)	Local Government Role and Levers Addressed	Partners and Funding Sources	Enablers, Barriers and Considerations
MacCartan and Palermo (2016) [34]	To examine how a food policy coalition influences a local food environment, focusing on coalition composition, functions, processes, strategies and policy outputs	Qualitative case study (Victoria)	Role: Coalition member; policy contributor; land use and institutional policy influencer Levers addressed: Policy advocacy: land use policy; institutional food policies; cross-jurisdictional coordination	Partners: LGs; health services; non-profit agencies; agriculture and food enterprises Funding: State government funding for convening agency; additional external grants (as available)	Enablers: Strong leadership; neutral convenor; LG participation; small membership Barriers: Limited funding; constrained formal authority; long policy timelines Considerations: Coalitions require facilitation, trust, and realistic expectations regarding policy change
Slade and Carter (2016) [36]	To analyse institutional fit and local government capacity in food security initiatives	Comparative qualitative case study (Victoria)	Role: Coordinating actor; policy implementer; constrained system steward Levers addressed: Policy development; organizational alignment; partnership governance	Partners: State government departments; LGs; community agencies Funding: VicHealth; Victorian Department of Health	Enablers: Clear role definition; leadership alignment Barriers: Neoliberal governance constraints; fragmented authority Considerations: Institutional fit must be planned explicitly across governance levels
Slade and Baldwin (2017) [35]	To critique inter-governmental partnership approaches supporting local government responses to food insecurity	Comparative qualitative case study (Victoria)	Role: Primary implementer; policy developer; integrator of food security into planning Levers addressed: Municipal public health planning: land use planning; transport planning; local infrastructure	Partners: State health agencies; LGs; evaluators Funding: VicHealth; Victorian Department of Health	Enablers: Senior leadership support; evidence collection; statutory planning cycles Barriers: Staff turnover; limited authority; planning legislation misalignment Considerations: Capacity-building requires realistic scope, stable staffing, and senior endorsement
Slade and Carter (2017) [37]	To examine how local governments operationalize social sustainability through food security	Qualitative policy document analysis + interviews (Victoria)	Role: Prioritize policy; service provider; equity advocate Levers addressed: Council plans; municipal wellbeing policies; food security policies	Partners: State agencies; community organizations Funding: State health promotion funding (VicHealth/DoH)	Enablers: Political acceptability of equity framing Barriers: Limited capacity to pursue full social sustainability agenda Considerations: LGs prioritize achievable outcomes under constraint
Thornton (2017) [50]	To explore governance tensions between community gardens and local councils	Qualitative case study and survey (Australian Capital Territory and New South Wales)	Role: Regulator; land use decision-maker; perceived gatekeeper Levers addressed: Zoning; land tenure; community garden policies	Partners: Community gardeners; councils; urban activists Funding: University research funding	Enablers: Community mobilization Barriers: Planning requirements; council priorities Considerations: Policy frameworks alone do not guarantee implementation
Cuttler et al. (2019) [47]	To measure healthy food costs across a Victorian region and examine price drivers	Repeated cross-sectional observational study (2014–2016) using the Victorian Healthy Food Basket (Victoria)	Role: LGs identified food access as a regional priority; LG staff supported data collection and interpretation Levers addressed: Planning powers to support supermarket competition; approval and incentives for new food retail outlets	Partners: Regional Food System Alliance; multiple LGAs; community health organizations Funding: Not reported	Enablers: Availability of standardized food basket tools; LG collaboration Barriers: Geographic isolation; limited competition Considerations: Data can inform LG planning, incentives, and regional food system interventions
Reeve et al. (2020) [46]	To analyse policies from six NSW local governments relevant to promoting a healthy food supply and consumer food environment	Qualitative policy document analysis (New South Wales)	Role: Policy developer; regulator; service provider; coordinator of food-related initiatives Levers addressed: Governance; food safety regulation; procurement; grants; education; land use planning (limited)	Partners: State food authorities; community organizations; food relief agencies Funding: Not reported	Enablers: Existing LG statutory responsibilities (food safety, waste); local policy autonomy Barriers: Limited planning authority; lack of state/federal nutrition policy frameworks Considerations: Dedicated whole-of-government food policies may improve coherence and impact
Riesenberg et al. (2020) [53]	To assess LG policies, attitudes and practices relating to obesity prevention and healthy food provision in LG-owned sporting facilities	Cross-sectional survey and policy document review of Victorian LGs (Victoria)	Role: Facility owner/operator; policy adopter; health promoter Levers addressed: Procurement; facility contracts; internal policies	Partners: VicHealth; Healthy Eating Advisory Service Funding: VicHealth (Water in Sport grant)	Enablers: Leadership support; funding; control over facilities Barriers: Limited resources; supplier issues; staff time Considerations: Contract clauses may be more feasible than high-level policies
Blake et al. (2021) [51]	To investigate changes in stakeholder commitment and perceptions during implementation of a healthy beverage policy	Longitudinal mixed-methods study (interviews, surveys, sales data, audits) (Victoria)	Role: Policy adopter; implementer; facility manager Levers addressed: Procurement; facility management; policy enforcement	Partners: Healthy Eating Advisory Service; academic researchers Funding: VicHealth; participating LGs	Enablers: Stepwise trials; dietitian support; stakeholder engagement Barriers: Revenue concerns; customer resistance; supply constraints Considerations: Allow time for adaptation; align health and financial priorities
Carrad et al. (2022) [41]	To systematically document LG food system policy actions	Policy mapping across 207 LGs (New South Wales and Victoria)	Role: Regulator; service provider; land manager; educator Levers addressed: Planning; food safety; waste; procurement; education	Partners: State agencies; community groups Funding: Not reported	Enablers: Legislative mandates (Victoria); waste responsibilities Barriers: Limited economic levers; planning constraints Considerations: Coherent, joined-up policies needed
Carrad et al. (2022) [33]	To examine food system policy development and implementation processes	Multiple qualitative case studies (focus groups) (New South Wales and Victoria)	Role: Convenor; policy developer; partnership broker Levers addressed: Planning; waste; economic development; education	Partners: NGOs; community groups; state agencies Funding: LG budgets	Enablers: Champions; leadership; dedicated staff Barriers: Funding instability; lack of state mandate Considerations: Dedicated roles and long-term funding critical
Love et al. (2022) [44]	To identify policy, programmatic and research opportunities to improve nutrition across the first 2000 days in Victoria	Rapid review, policy mapping and qualitative stakeholder interviews (Victoria)	Role: Implementation of Municipal Public Health and Wellbeing Plans; coordination of early years nutrition initiatives Levers addressed: Statutory planning (MPHWPs); service coordination; procurement; advocacy	Partners: State government, LGs, NGOs, health and education sectors Funding: VicHealth (commissioned research)	Enablers: Existing statutory frameworks; systems approach familiarity Barriers: Fragmented policies; lack of regulation; limited funded capacity Considerations: Need for funded coordination roles and joined-up governance
Riesenberg et al. (2022) [54]	To assess LG policies, priorities and practices relating to obesity prevention and healthy food in sport and recreation facilities	Cross-sectional national survey of Australian LGs (Victoria)	Role: Owner/operator of sport and recreation facilities; policy developer Levers addressed: Facility food policies; procurement; lease conditions	Partners: State governments; NGOs; universities Funding: VicHealth (Water in Sport Initiative)	Enablers: Stakeholder support; control over facilities; funding Barriers: Not reported Considerations: Limited control; financial viability concerns; lack of policy mandates
Barbour et al. (2023) [32]	To explore the way contextual factors either facilitate or impede the policymaking process to support healthy and sustainable diets	Qualitative case study using semi-structured interviews (Victoria)	Role: Policy leader; convenor; implementer Levers addressed: Food system strategy; partnerships; advocacy; workforce investment	Partners: Community groups; neighbouring LGs; state and global networks (e.g., UNESCO) FUNDING LG operational funding	Enablers: Leadership; partnerships; workforce capacity; evidence use BARRIERS Funding insecurity; higher-level policy misalignment; COVID-19 Considerations: Long-term commitment; slow-burn approach; sustained partnerships
Carrad et al. (2023) [38]	To explore food system issues addressed by Australian local governments; identify policies, programs, governance arrangements, enablers and barriers	Cross-sectional online survey of LGs (n = 64) (New South Wales and Victoria)	Role: Policy maker, service provider, planner, convener, regulator Levers addressed: Food waste, events, potable water, procurement, planning, community development, partnerships	Partners: LGs; state government agencies; community organizations; universities Funding: Not reported	Enablers: Internal LG support; staff capacity; partnerships; external funding; local data Barriers: Limited human resources; funding constraints; planning legislation; lack of mandate Considerations: Legislative reform and sustained resourcing needed to enable LG food system action
Dangerfield et al. (2023) [42]	To examine local government actions related to food environments as documented in Municipal Public Health and Wellbeing Plans (MPHWPs)	Documentary policy analysis of MPHWPs (Victoria)	Role: Policy planner and implementer via statutory MPHWPs Levers addressed: Organizational food environments (council-run facilities); limited land use/zoning action due to state constraints	Partners: Not systematically reported Funding: Not reported	Enablers: Greater LG capacity in organizational settings Barriers: State legislative constraints; lack of monitoring frameworks Considerations: Need for clearer indicators, environmental-level measures, and evaluation guidance
Day et al. (2023) [48]	Assess public health nutrition workforce capacity to work with food retail	Convergent mixed-methods empirical study (Northern Territory)	Role: Indirect: system actor shaping workforce roles Levers addressed: Workforce structures; mandates; funding	Partners: ALPA; Indigenous & health services Funding: Not reported	Enablers: Community relationships; experienced workforce Barriers: Medical model dominance; limited tools Considerations: System-level reform needed for co-design
Molenaar et al. (2023) [49]	To explore community members’ experiences of food access and food insecurity to inform local government responses	Sequential mixed-methods study (survey and photovoice interviews) (Victoria)	Role: Policy developer and implementer (via Community Food Strategy) Levers addressed: Community food strategy; engagement; food access planning	Partners: Cardinia Shire Council; Monash University Funding: Monash–Warwick Alliance Catalyst Grant	Enablers: Community participation; local strategy; food literacy Barriers: Cost of living; transport; limited retail options; reliance on food relief Considerations: Need to embed lived experience in LG policy and program design
Blake et al. (2025) [52]	Assess impact of LG healthy drinks capacity-building intervention on policies, priorities, and stakeholder support	Non-randomized mixed-methods evaluation; repeat cross-sectional with controls (Victoria)	Role: Policy owner, implementer, facilitator within LG facilities Levers addressed: Policy; procurement; retail standards; staffing; regulation	Partners: LGs; VicHealth; facility managers Funding: VicHealth	Enablers: High LG readiness; dedicated staff; state alignment Barriers: Ceiling effects; reliance on LG ownership Considerations: Capacity-building works where readiness exists
Godrich et al. (2025) [40]	To understand how organizations partner to support food security in rural, regional and remote areas	Qualitative interviews (n = 94 interviews; 101 participants) (Western Australia)	Role: Partner, funder, coordinator, policy aligner Levers addressed: Partnerships; funding; service coordination; community engagement	Partners: LG; NGOs; Aboriginal organizations; businesses; state government Funding: Not reported	Enablers: Trust-based relationships; shared resources; local knowledge Barriers: Funding insecurity; workforce turnover; lack of formal agreements Considerations: Need for clearer roles, documentation, and sustainable resourcing
Godrich et al. (2025) [39]	To identify systems-change capacity of food security initiatives	Qualitative systems-change case study (Western Australia)	Role: Actor within ecosystem Levers addressed: Policy alignment; advocacy interfaces	Partners: Universities; government; community sector Funding: Western Australian Government	Enablers: Grassroots innovation and networks Barriers: Weak government–community linkage Considerations: Community action alone insufficient
Keegan et al. (2025) [43]	To examine how sub-national policies support food security and resilience	Comparative interpretive policy analysis (New South Wales and Queensland)	Role: Planner and implementer; limited mandate Levers addressed: Planning; emergency management; community development	Partners: None (document-based) Funding: Public research funding	Enablers: Strong agricultural policy foundations Barriers: Fragmented governance; weak food access focus Considerations: Requires integrated multi-level governance
Pickles et al. (2025) [45]	To map and assess role of home/community gardening in local food system resilience	Mixed-methods situational analysis (New South Wales)	Role: Landowner, facilitator, potential policy enabler Levers addressed: Land access; urban planning; grants	Partners: Councils (indirect); community organizations Funding: Not reported	Enablers: Strong community organizations; peer learning Barriers: Land insecurity; limited LG support Considerations: Needs bottom-up and top-down support

**Table 4:** Outline of included grey literature documents (n = 15).

Author (Year)	Document Type and Intended Use	Scope	Food System/Food Environment Focus	Actions/Strategies Described	Local Government Roles and Partnerships	Implementation Considerations
Heart Foundation Tasmania (2016) [69] ^+^	Local government situational analysis/community food access profile: Clarence Council Area	Clarence City Council LGA, Tasmania (urban–peri-urban) Population: Whole population; emphasis on low-income households, people with disability, older residents, and car-limited households.	Retail distribution across suburbs; affordability; farmers’ markets; food relief hubs; spatial access	Community food hubs; social eating programs; local markets; gardens; cooking and budgeting programs	Roles: LG as supporter of community hubs, markets, gardens, and coordination of partners Partnerships: Heart Foundation Tasmania; UTAS; Food Connections Clarence; schools; neighbourhood houses; NGOs	Spatial inequities across suburbs; transport and income constraints
Heart Foundation Tasmania (2016) [68] ^+^	Local government situational analysis/community food access profile: Devonport City Council Area	Devonport City Council (Tasmania) Population: Whole community, with identification of vulnerable groups (low-income households, people without cars, people with disability, single-parent households)	Physical and economic access to healthy food; affordability; retail availability; transport	Food security networks; farmers’ markets; food box schemes; community programs	Roles: Supportive role in planning, coordination, and local initiatives Partnerships: Community organizations, food security networks, council–community partnerships	Transport access, income constraints, geographic inequities
Heart Foundation Tasmania (2016) [67] ^+^	Local government situational analysis/community food access profile: Dorset Council Area: Community/Household	Dorset Council (Tasmania) Population: Whole community, with emphasis on low-income households, people without transport, families, older adults (Tasmania)	Food access, affordability, retail availability, local markets	Community markets; food cooperatives; cooking and food literacy programs	Roles: Planning support; partnership facilitation; integration with health and wellbeing activities Partnerships: Community houses, retailers, health services, council	Transport, geographic isolation, income
Heart Foundation Tasmania (2016) [56] ^+^	Local government situational analysis/community food access profile: Glamorgan Council Area	Glamorgan–Spring Bay Council (rural/regional) (Tasmania) Population: Whole population; strong focus on low-income households, households without cars, and geographically isolated residents	Limited retail availability; affordability; transport to shops; farmgate sales and community markets	Farmgate sales; markets; school and community gardens; healthy eating promotion	Roles: LG as facilitator of community initiatives and economic development linkages Partnerships: Heart Foundation Tasmania; UTAS; schools; community markets; local producers	Geographic dispersion; limited retail options; transport constraints
Heart Foundation Tasmania (2016) [55] ^+^	Local government situational analysis/community food access profile: Glenorchy Council Area	Glenorchy City Council (urban, socio-economically disadvantaged areas) (Tasmania) Population: General population; priority focus on low-income households, single parents, people without cars, and people with poor health	Retail mix; affordability; markets; community food programs; geographic inequities	Community and school gardens; markets; healthy catering; food skills programs; food relief	Roles: LG as facilitator and supporter; reference to embedding activities into long-term council plans Partnerships: Heart Foundation Tasmania; UTAS; MONA 24 Carrot Gardens; schools; food relief agencies; NGOs	High disadvantage; transport access; reliance on community-based delivery.
Heart Foundation Tasmania (2016) [66] ^+^	Local government situational analysis/community food access profile: Hobart City Council Area	Hobart City Council LGA (urban) (Tasmania) Population: General population; priority focus on low-income households, single-parent families, older people, people with disability, and households without cars	Retail availability of fruit and vegetables; affordability; transport access; markets and community food outlets	Supporting markets; healthy catering; community and school gardens; food skills programs; food relief distribution	Roles: LG positioned as coordinator and supporter of local initiatives; no regulatory or statutory actions specified Partnerships: Heart Foundation Tasmania; University of Tasmania; markets; food relief agencies; schools; community groups	Transport access; cost of food; coordination across existing programs rather than new interventions.
Heart Foundation Tasmania (2016) [65] ^+^	Local government situational analysis/community food access profile: Huon Valley Council Area	Huon Valley Council (regional/rural) (Tasmania) Population: Whole population; emphasis on low-income households, people without cars, and dispersed rural communities	Retail and market access; farmgate sales; affordability; resilience factors	Markets; farmgate sales; social enterprises; community food skills programs	Roles: LG as supporter of producers, community enterprises, and coordination of local initiatives Partnerships: Heart Foundation Tasmania; UTAS; Huon Producers Network; community houses; schools	Long travel distances; reliance on local producers; seasonal variability.
Heart Foundation Tasmania (2016) [64]	Local government situational analysis/community food access profile: Flinders Island Council Area	Flinders Island Council area, including Whitemark, Lady Barron, Cape Barren Island, and surrounding island group (remote island LGA) (Tasmania) Population: Entire island population, with explicit focus on low-income households, single-parent families, children in poverty, people with disability or chronic disease, households without cars, and residents experiencing geographic isolation	Physical and economic access to healthy food; limited retail availability; transport and freight supply chains; community markets; home food production; affordability constraints specific to island contexts	Community markets; support for local growers and home food production; school kitchen gardens (Stephanie Alexander program); meals on wheels and food delivery; promotion of locally grown food (Flinders Island Fresh); healthy catering; cooking and food skills programs	Roles: Council role in monitoring food transport and cold-chain integrity; purchasing infrastructure (e.g., containers) to maintain food quality; supporting food safety, health promotion, and coordination of local initiatives Partnerships: Heart Foundation Tasmania; University of Tasmania; Flinders Island District High School; Rural Primary Health Service; food relief agencies; community markets; transport and freight providers	Extreme remoteness; limited retail outlets; reliance on air and sea freight; weather-related disruptions; cold-chain management; small population size; capacity constraints
Tasmanian Department of Health and Human Services (2016) [63]	State-level consultation and evaluation report to inform policy and funding	Tasmania (state-wide) Population: Community organisations, social enterprises, LG, funders, researchers	Food security; local food systems; food access and affordability; community food initiatives	Community gardens, food hubs, markets, food literacy programs, social enterprises	Roles: Broker, facilitator, partner in local food systems and community-led solutions Partnerships: Strong emphasis on cross-sectoral and whole-of-government collaboration	Sustainability, funding duration, partnership management, volunteer burnout
North East Local Food Strategy Working Group (2019) [62]	Regional strategy and planning framework; strategic resource to guide coordinated action and support funding applications	North East Victoria, covering the LGAs of Alpine, Benalla, Indigo, Mansfield, Towong, Wangaratta and Wodonga (Victoria) Population: Regional communities, food producers, LGs, health services, community organizations	Whole food system: production; distribution and processing; consumption (food literacy, access, food choice); waste reduction	Strategic directions and priority actions across five themes: leadership & advocacy; production; distribution & processing; consumption; waste reduction	Roles: Policy development; land use planning; food procurement; advocacy; community engagement; coordination across councils Partnerships: Explicit cross-sector governance involving health, LG, agriculture, tourism, natural resource management and community sectors	Need for coordinated regional leadership; funding acquisition; scale-appropriate logistics and planning reforms
Latrobe Health Assembly (2021) [61]	Program report; monitoring and accountability document	Latrobe Valley (Victoria) Population: Latrobe Valley community, with focus on people experiencing food insecurity	Food security; access; local food systems; food literacy; emergency food relief	Community interventions; food relief coordination; education campaigns; food system mapping	Roles: Partner and facilitator within a multisector coalition Partnerships: Strong multisector governance model (health, LG, NGOs, community, retailers)	Capacity, collaboration, COVID-19 disruption
The Sax Institute (2021) [60]	Planning tool/discussion framework; visual poster series designed to support local food security planning	Urban and regional Aboriginal communities (National, Australia) Population: Aboriginal communities, Aboriginal community organizations, local councils and health services	Food security; retail food environment; food affordability and availability; food relief; transport and housing access; cultural food knowledge	Structured prompts across domains (retail environment, food relief, education, transport, housing, leadership) to guide community-led planning	Roles: Local councils identified as potential convenors and partners in planning and systems change Partnerships: Central emphasis on strong Aboriginal leadership, partnerships, and shared decision-making	Systems-based approach; cultural appropriateness; community-led use
Bendigo Health-Loddon Mallee Public Health Unit (2024) [59]	Project brief/monitoring and reporting document	Buloke, Gannawarra, Swan Hill and Campaspe LGAs (Victoria) Population: Community members, organizations, and primary producers	Local food systems; food security; food literacy; local food production	Capacity-building workshops; food systems mapping; survey dissemination	Roles: LGAs identified as key stakeholders and beneficiaries of capacity building Partnerships: Project Reference Group with geographic and sector representation	Small sample size; results not generalizable; need for further LGA-specific analysis
Food Connect Foundation (2025) [57]	National strategy, case study, and investment roadmap; intended to support replication of food hubs and inform governments and funders.	National (Australia), with regional and local case studies Population: Regional producers (“missing middle” farmers), food enterprises, communities, and LGs	Regional food systems; food hubs; infrastructure for aggregation, processing, distribution; regenerative and resilient supply chains	Establishing food hubs; building shared infrastructure; replication pathways; procurement reform; blended finance models	Roles: LG as enabler, land partner, procurement actor, planning authority, and co-investor in infrastructure. Partnerships: Multi-level governance; cross-sector partnerships; Indigenous governance; community ownership models; collaboration with NGOs, investors, and institutions	Capital requirements; governance models; regulatory alignment; community readiness; scaling constraints
Brimbank City Council (2025) [58]	Statutory Municipal Public Health and Wellbeing “scan”; intended to inform MPHWP priorities and council-wide planning.	Brimbank City Council (metropolitan Melbourne) (Victoria) Population: Whole population, with emphasis on priority populations experiencing disadvantage (e.g., low income, culturally and linguistically diverse communities, people with disability)	Food insecurity, healthy eating, diet-related chronic disease, exposure to unhealthy food environments (e.g., sugary drinks, retail proximity)	Strategic responses aligned to state priorities; advocacy; regulatory influence (where relevant); partnership-based interventions	Roles: Statutory planner, policy implementer, advocate, and partner responsible for population health improvement Partnerships: Explicit alignment with Victorian Public Health and Wellbeing Act; partnerships with Western Public Health Unit, VicHealth, state agencies, NGOs, and community organizations	Legislative mandates; cross-council integration; data gaps; resource constraints; regional coordination

^+^ Document series/source program: Seven included grey literature documents were council-level profiles produced through the Healthy Food Access Tasmania initiative. They were retained as separate documents because they reported distinct local contexts; however, they formed a related document series using a common program and reporting approach and should not be interpreted as seven independent evaluations.

## Data Availability

The original contributions presented in this study are included in the article/Supplementary Material. Further inquiries can be directed to the corresponding author.

## Supplementary Materials

The following supporting information can be downloaded at https://www.mdpi.com/article/10.3390/nu18162611/s1: Supplementary Table S1: Peer-reviewed search strategy. Supplementary Table S2: Grey literature search strategy. Supplementary Table S3: Outline of included peer-reviewed articles (n = 23). Supplementary Table S4: Outline of included grey literature documents (n = 15). Supplementary Table S5: Theme-specific coding guide for mapping included studies and reports to local governments’ healthy food environment research priorities. Supplementary Table S6: Rationale for mapping included studies and reports to local governments’ healthy food environment research priorities.

## Abbreviations

The following abbreviations are used in this manuscript:

LG	Local Government
LGA	Local Government Area
NGO	Non-government Organization
