# Supplementary material for: Supporting Healthier, Sustainable and Equitable Food Environments: A Rapid Review of Australian Local Government Action
Source: Nutrients. 2026 Aug 10;18(16):2611. doi: 10.3390/nu18162611 (PMC13516812; doi:10.3390/nu18162611)
Supplement: Supplementary file 1 [file nutrients-18-02611-s001.zip › nutrients-4362677-supplementary.pdf]

Table S1. Peer-reviewed search strategy

Concept 1	“Local Government" OR "local government*" OR council* OR municipal* OR "local authorit*" OR "local planning" OR shire OR “local authority” OR “local council” OR county OR municipality OR region OR district OR city OR town OR township OR borough
AND	
Concept 2	"(food OR built OR organisational OR community OR consumer OR information) AND environment*" OR "food supply" OR "food systems" OR "food retail*" OR "food system*" OR "healthy food*" OR "food access" OR "food availab*" OR "food secur*"
AND	
Concept 3	"urban planning" OR "zoning" OR polic* OR strateg* OR intervention* OR program* OR governance OR planning OR regulation*
AND	
Concept 4	Australia* OR "New South Wales" OR Victoria OR Queensland OR Tasmania OR "South Australia" OR "Western Australia" OR "Australian Capital Territory" OR "Northern Territory"

Table S2. Grey literature search strategy

**Strategy**	**Search string(s)**	**Websites**
Grey literature databases	“local government” AND “food environment” AND Australia	• Trove (National Library of Australia) - research & reports: https://trove.nla.gov.au<br>• Australian Policy Observatory - https://apo.org.au/collections
	council AND “food strategy”	
	“municipal public health” AND food	
Customised Google search engines[#]	site:gov.au "food environment" AND council filetype:pdf	• Google (advanced operators): https://www.google.com
	site:org.au "healthy food" AND local government filetype:pdf	

All Google-based queries were conducted using an incognito browsing window to minimise the influence of personalised search histories, cookies, or browser stored preferences on the retrieval of search results

Table S3. Outline of included peer-reviewed articles (n = 23)

Authors (year)	Title	Intervention / policy name	Key characteristics	Outcomes reported	Geographic context	Sociodemographic context
McCartan and Palermo (2017)	The role of a food policy coalition in influencing a local food environment: an Australian case study	Local food policy coalition (no formal program name specified)	Small core membership; cross-sector representation; neutral convening organisation; collaborative advocacy focus	Increased collaboration; improved policy advocacy capacity; contribution to local policy changes (eg gardens on LG land)	Rural/regional Victoria, Australia	Rural communities; agriculture- and tourism-based local economies
Slade and Carter (2016)	Towards institutional fit? The reality of institutional capacity through two food security exemplars	Food for All; Food Security and Access Policy Development Project	Capacity-building partnerships; focus on internal–external alignment	Improved short-term organisational capacity; limited sustained change	Victoria, Australia	Disadvantaged urban and rural LGAs
Slade and Baldwin (2017)	Critiquing Food Security Inter-governmental Partnership Approaches in Victoria, Australia	Food for All (P1); Food Security and Access Policy Development Project (P2)	State-led funding; LG project officers; integrated planning requirement; policy development focus	Short-term capacity gains; increased policy visibility; variable institutionalisation	Victoria, Australia	Low-socioeconomic and culturally diverse municipalities
Slade and Carter (2017)	Local Governance for Social Sustainability: equity as a strategic response to neoliberal constraints in food security initiatives	Municipal food security and health promotion initiatives	Strategic focus on equity; selective goal setting	Equity embedded in LG policy discourse	Victoria, Australia	High-disadvantage, CALD and urban growth LGAs
Thornton (2017)	"The Lucky country"? A critical exploration of community gardens and city–community relations in Australian cities	Community gardens (various local initiatives)	Grassroots-led; informal governance; contested approvals	Tension between councils and communities	Sydney and Canberra, Australia	Mixed-income urban communities
Cuttler et al (2019)	An investigation of the cost of food in the Geelong region of rural Victoria: Essential data to support planning to improve access to nutritious food	Not an intervention; monitoring and assessment activity	Use of Victorian Healthy Food Basket across multiple LGAs	Higher healthy food costs associated with distance from regional centre, lack of competition, and independent stores	Five rural LGAs in the G21 region, Victoria	SEIFA measured; no association found
Reeve et al (2020)	The role of Australian local governments in creating a healthy food	Multiple LG food- and nutrition-related policies (no single intervention)	Policies dispersed across multiple non-food-specific documents; limited	Identification of food system actions and gaps in LG policy coverage	Sydney metropolitan region, NSW, Australia	Mix of advantaged and disadvantaged urban LGAs

	environment: an analysis of policy documents from six Sydney local governments		dedicated nutrition policies			
Riesenberg et al (2020)	Policies influencing the provision of healthy food and drinks in local government-owned sport and recreation facilities in Victoria, Australia	Healthy food and drink provision policies (varied by LG)	Focus on beverages and food sold in LG-owned facilities	Proportion of LGs with policies; extent of food environment changes	Victoria, Australia	Diverse LGAs; higher SE LGs more advanced
Blake et al (2021)	Implementing a healthy food retail policy: a mixed-methods investigation of change in stakeholders' perspectives over time	Healthy Choices Guidelines implementation	Stepwise implementation; beverage-focused	Increased stakeholder optimism; improved beverage availability	Metropolitan Melbourne, Victoria	LGs in relatively disadvantaged areas
Carrad et al (2022)	Australian local government policies on creating a healthy, sustainable, and equitable food system: analysis in New South Wales and Victoria	Food system policies and related LG actions	Actions dispersed across many policy domains	Frequency of LG action by policy domain	NSW and Victoria, Australia	Metropolitan and non-metropolitan LGs
Carrad et al (2022)	Local innovation in food system policies: A case study of six Australian local governments	LG food system strategies (varied by case)	Place-based; multi-sectoral; participatory	Description of implementation activities	NSW and Victoria	Urban, peri-urban and rural LGs
Love et al (2022)	A call for joined-up action to promote nutrition across the first 2000 days of life using a food systems approach	No interventions; mapping of opportunities	Systems-based, multi-sectoral early-years nutrition focus	Identification of policy gaps (external food environment, supply chains); priority opportunities	Victoria, Australia	Early childhood populations; equity considerations noted
Riesenberg et al (2022)	Local government policies on healthy food promotion and obesity prevention: results from a national Australian survey	Healthy food and drink policies in LG-owned facilities	Mostly voluntary policies; uneven adoption	Only 10% of LGs had formal policies; more action in larger, urban LGs	National (all Australian states and territories)	Variation by SEIFA, population size, remoteness
Barbour et al (2023)	Perseverance, partnerships and passion: ingredients for successful	Greater Bendigo Food System Strategy (2020–2030)	Systems-based, integrated food policy	Successful development of evidence-based food system policy	City of Greater Bendigo, regional Victoria	Regional population (~120,000); agricultural region

	local government policy to promote healthy and sustainable diets					
Carrad et al (2023)	Food system policy making and innovation at the local level: Exploring the response of Australian local governments to critical food systems issues	Multiple LG-led initiatives; no single intervention	Wide variation in LG action; stronger engagement in Victoria than NSW	Types of food system activities; governance processes; perceived barriers and enablers	New South Wales and Victoria, Australia	Metropolitan and non-metropolitan LGs
Dangerfield et al (2023)	Planning healthy food environments: An analysis of local government municipal public health and wellbeing policy in regional Victoria	Municipal Public Health and Wellbeing Plan (2019–2023)	Actions concentrated in organisational settings (sporting clubs, workplaces, council facilities)	Presence/absence of actions across food environment domains; lack of measurable indicators	Victoria, Australia	Regional health inequality acknowledged; not analysed quantitatively
Day et al (2023)	Towards healthy food retail: An assessment of public health nutrition workforce capacity to work with stores	Not an intervention (capacity assessment)	Interviews, surveys, service mapping	Identified workforce capacity gaps	NT & Far North QLD (remote)	Aboriginal and Torres Strait Islander communities; remote
Molenaar et al (2023)	Harnessing community expertise in understanding food insecurity to inform responses in a local government area in Victoria, Australia: a mixed methods photovoice study	Cardinia Shire Community Food Strategy (2018–2026)	Emphasis on lived experience; participatory co-production of knowledge	Themes describing food access, food insecurity drivers, coping strategies	Cardinia Shire, Victoria, Australia	Adult community members; mix of food secure and food insecure households
Blake et al (2025)	Changes in Local Government Priorities and Stakeholder Satisfaction Following a Healthy Drinks Capacity-Building Intervention in Sports and Recreation Facilities in Victoria, Australia	*Water in Sport* (WIS) initiative	LG project officers; nudges; two-year delivery; state-aligned	Policy adoption; stakeholder support; LG priorities	Victoria, Australia	Mixed LG populations; varied SEIFA

Godrich et al (2025)	Increasing the Effectiveness of Rural, Regional and Remote Food Security Initiatives Through Place-Based Partnerships—A Qualitative Study	Multiple food security initiatives (no single intervention)	Predominantly informal, relational partnerships	Types of partnership activities and modes of collaboration	Rural, regional and remote Western Australia	Diverse organisations; Aboriginal-led and non-government sectors
Godrich et al (2025)	Food security systems change: a case study from rural, regional, and remote Australia	*Food Community* systems-change project	Six-stage SIL process; 148 initiatives mapped	Systems-change capability profiles	Regional, rural & remote WA	Dispersed RRR populations
Keegan et al (2025)	Informing Governance for Food System Resilience: A Comparative Case Policy Analysis of Two Australian Regions Impacted by Multiple Disasters	Not applicable (policy landscape)	146-document analysis; multi-level	Policy strengths, gaps, integration levels	Northern Rivers NSW; Far North QLD	Disaster-prone, socioeconomically vulnerable regions
Pickles et al (2025)	Informing Governance for Food System Resilience: A Comparative Case Policy Analysis of Two Australian Regions Impacted by Multiple Disasters	None (observational study)	Garden mapping; surveys; thematic analysis	Gardening outputs, motivations, barriers	Illawarra–Shoalhaven NSW	Urban- peri-urban; income and land-access inequity

Table S4. Outline of included grey literature documents (n = 15)

Author (year)	Title	Data sources referenced	Monitoring indicators proposed	Evaluation or review	Equity framing
Heart Foundation Tasmania (2016)	Clarence Council Area–Community/Household Food Access Profile	Healthy Food Access Basket Survey; Tasmanian Population Health Survey; ABS; Social Health Atlas	Food basket affordability; outlet mapping; participation in food programs	No formal evaluation; descriptive and planning-oriented	Strong focus on disadvantage, food insecurity, and access barriers
Heart Foundation Tasmania (2016)	Devonport City Council Area–Community Food Access Profile	Healthy Food Access Basket Survey; population health survey data; outlet mapping; administrative data	Yes – food basket cost data, outlet availability, population indicators	Monitoring data reported; no formal evaluation of initiatives	Explicit
Heart Foundation Tasmania (2016)	Dorset Council Area–Community/Household Food Access Profile	Healthy Food Access Basket Survey; population data; outlet mapping	Yes – basket cost data, access indicators	Descriptive monitoring only; no outcome evaluation	Explicit
Heart Foundation Tasmania (2016)	Glamorgan Council Area–Community/Household Food Access Profile	Healthy Food Access Basket Survey; Tasmanian Population Health Survey; ABS Census	Availability and price of healthy food; number of outlets; diet indicators	None specified	Strong focus on rural disadvantage and access inequity
Heart Foundation Tasmania (2016)	Glenorchy Council Area–Community/Household Food Access Profile	Healthy Food Access Basket Survey; Population Health Survey; ABS; UTAS research	Retail availability; food basket cost; diet indicators	No evaluative framework; baseline mapping only	Explicit, with strong emphasis on disadvantage and social determinants
Heart Foundation Tasmania (2016)	Hobart City Council Area–Community/Household Food Access Profile	UTAS Healthy Food Access Basket Survey (2014); Tasmanian Population Health Survey; Social Health Atlas; ABS Census; TFARC research	Healthy food basket cost; number/type of outlets; self-reported diet indicators (fruit/vegetable intake)	No evaluation framework; intended as a baseline snapshot	Explicit focus on food insecurity, poverty, and social disadvantage
Heart Foundation Tasmania (2016)	Huon Valley Council Area–Community/Household Food Access Profile	Healthy Food Access Basket Survey; Population Health Survey; ABS; TFARC	Food basket cost; outlet availability; diet indicators	No evaluation framework specified	Explicit focus on food insecurity and rural disadvantage
Heart Foundation Tasmania (2016)	Flinders Island Council Area–Community/Household Food Access Profile	Tasmanian Population Health Survey; Social Health Atlas of Australia; ABS Census data; UTAS Healthy Food Access Basket Survey (explicitly notes no local data collection	Descriptive indicators only: number of food outlets; transport access; diet indicators (fruit/vegetable intake); population vulnerability characteristics.	No formal evaluation framework; document functions as a descriptive baseline profile	Strong and explicit equity focus on food insecurity, poverty, transport barriers, disability, and island-specific disadvantage

		due to logistics); TFARC literature	No local food basket cost data available.		
Tasmanian Department of Health and Human Services (2016)	Food Security Consultation 2016: Building on what is working and future directions	Semi-structured interviews (n=25); stakeholder consultation; policy documents	Monitoring discussed (e.g. population health surveys, food basket data), not directly implemented	Qualitative evaluation using thematic analysis; ethics approval reported	Explicit (disadvantaged communities, food insecurity, access barriers)
North East Local Food Strategy Working Group (2019)	North East Local Food Strategy 2018–2022: Strengthening the local food system in North East Victoria	Secondary data (ABS, DHHS, Tourism North East); prior feasibility studies; food mapping projects; community consultations and regional forums	No formal monitoring framework reported; reliance on existing population health, agriculture and waste data sources	No evaluation of the Strategy reported; intended to guide future project development and implementation	Explicit (food access, food security, social determinants of health, vulnerable populations)
Latrobe Health Assembly (2021)	Latrobe Food Security Coalition: Impact Report 2021	Program monitoring data; food security surveys; food basket pricing; systems mapping	Yes – participation numbers, population reach, food basket cost trends	Process and monitoring data reported; no causal effectiveness evaluation	Explicit
The Sax Institute (2021) 35]	Murradambirra Dhangaang (make food secure): a food security planning tool for Aboriginal communities in urban and regional areas	Research undertaken by the SEARCH team; adaptation of the Good Food Planning Tool (Brimblecombe et al, 2015)	No formal indicators; reflective and qualitative prompts used instead	No evaluation reported for the tool itself	Explicit and central (Aboriginal leadership, cultural knowledge, self-determination)
Bendigo Health - Loddon Mallee Public Health Unit (2024)	Murray Healthy Food Systems	Community food and farming survey (n=200); workshop participation data; post-workshop evaluation	Yes – participation counts; survey indicators; self-reported learning outcomes	Post-workshop evaluation (self-reported knowledge and awareness)	Implicit (food insecurity prevalence, cost-of-living pressures)
Food Connect Foundation (2025)	Regenerating the regions - how food hubs can build resilience	Stakeholder interviews; case studies; SROI analyses; international food hub literature; investment and procurement research	Social return on investment; number of enterprises supported; infrastructure utilisation; food access outcomes (illustrative)	Impact assessment discussed (SROI); reflective learning from case studies rather than formal trials	Explicit focus on community ownership, Indigenous epistemologies, equitable supply chains, and access to affordable food
Brimbank City Council (2025)	Health and Wellbeing Profile 2025	Victorian Population Health Survey; ABS Census; PHIDU Social Health Atlas; DHHS datasets; Cancer Council Victoria; WPHU spatial analyses	Diet indicators (fruit/veg intake, sugary drinks); food insecurity prevalence; chronic disease rates; spatial exposure indicators	Ongoing population monitoring tied to four-year MPHWP cycle	Strong, explicit equity lens using Fair Foundations, Environments for Health, intersectionality, and gender analysis

Table S5. Theme-specific coding guide for mapping included studies and reports to local governments' healthy food environment research priorities

Theme	Definition	Theme-specific inclusion	Theme-specific exclusion	Apply global coding rule after theme fit is established[#] (P, S, N)[+]
1. Urban planning strategies, tools and guidelines	Local government use, analysis or proposed use of planning, zoning, development controls, design standards, infrastructure siting, land tenure, transport or spatial-planning instruments to shape food access, outlet location, food production or food-system infrastructure.	Zoning and development controls; land-use policy; planning schemes; municipal planning instruments; siting or approval of food outlets, gardens, markets or food hubs; land access or tenure linked to LG planning; spatial planning recommendations tied to LG decisions.	Descriptive mapping without analysis of a planning instrument; generic references to planning; organisational procurement; council health planning with no built-environment or land-use mechanism.	**P:** central aim, intervention or policy domain; removing it changes the document purpose. **S:** meaningful supporting lever, context, recommendation or implication. **N:** absent, passing, generic or inferred only.
2. Evidence-based interventions for healthy food environments	Policies, programs or actions intended to change food-environment conditions where the document reports an intervention rationale, implementation, evaluation or empirical outcomes.	Implementation or evaluation of healthy food or drink policy; procurement or retail standards; capacity-building interventions; program actions with outcome or process data; policy implementation studies.	Policy mapping with no intervention implementation; descriptive monitoring only; recommendations for future action; strategies listing actions without implementation or evaluative evidence.	**P:** intervention design, implementation, acceptability, process or outcomes are central. **S:** intervention content is meaningful but not central. **N:** only future, descriptive, aspirational or inferred relevance is present.
3. Community engagement and co-design	Active involvement of communities, people with lived experience, priority populations or stakeholders in identifying problems, generating knowledge, setting priorities, designing actions, governing implementation or interpreting findings.	Co-design; co-production; photovoice; community-led planning; participatory workshops; shared decision-making; community ownership; Aboriginal-led approaches; stakeholder consultation that shapes findings or strategy.	Communities as recipients or survey respondents only; consultation merely acknowledged; program participation without influence over design or decisions; organisational partnerships without community involvement.	**P:** participatory, community-led or co-design methods are central. **S:** engagement is meaningful but supporting. **N:** involvement is absent, passive, tokenistic or only inferred.
4. Governance and multisectoral partnerships	Structures, roles, relationships and decision processes through which LGs work internally or with other sectors to coordinate food-environment or food-system action.	Coalitions; cross-sector governance; intergovernmental collaboration; institutional roles; partnership functioning; policy integration; coordination mechanisms; leadership and accountability structures.	Lists of partners or funders without role description or analysis; incidental collaboration; advocacy considered only as influence on higher government.	**P:** governance, institutional roles, partnership processes or coordination are central. **S:** governance provides meaningful context or an implementation condition. **N:** only named, implied or generic.
5. Knowledge translation and dissemination	Planned processes or products that convert research, monitoring data or practice knowledge into accessible information, tools, guidance, learning or decision support.	Profiles; dashboards; guidance; planning tools; practice codification; dissemination strategies; capacity-building for evidence use; collective learning; decision-support resources; action-oriented reporting.	Routine publication of findings; policy implications in a discussion section; data collection without a translation product or process; knowledge generated only for academic purposes.	**P:** translation, dissemination, practical guidance or decision support is the main purpose. **S:** knowledge sharing is a meaningful supporting mechanism. **N:** no distinct translation process or product is reported.
6. Food environment monitoring	Systematic collection, analysis, comparison or reporting of indicators describing food-environment	Food basket surveys; outlet audits and mapping; repeated measures; population or policy indicators; monitoring frameworks; routine	One-off qualitative description with no defined indicators; community views only; food-security experience measures without environmental	**P:** monitoring, indicator development, audit, mapping or repeated assessment is a main purpose or major output.

	conditions, policies or changes over time.	reporting; intervention monitoring using environment or policy measures.	monitoring; recommendations to develop monitoring with none undertaken.	**S:** monitoring is embedded in another activity.<br>**N:** no monitoring system, indicators or substantive assessment.
7. Food systems and food security	Understanding or acting on interconnected production, distribution, retail, access, consumption and waste systems, or on reliable, dignified access to sufficient, safe, nutritious and culturally appropriate food.	Food insecurity and access; affordability; resilience; supply chains; regional food systems; food hubs; food relief situated in a system; integrated production-to-waste strategies; food-system governance.	Narrow healthy-retail or organisational interventions with no systems or food-security framing; isolated dietary behaviour outcomes; generic use of the term food system.	**P:** food security, food access, resilience or whole-of-system action is central.<br>**S:** systems or food-security content meaningfully contextualises another focus.<br>**N:** only generic, passing or inferred.
8. Advocacy and local government powers	LG use, analysis or proposed use of statutory authority, regulatory powers, procurement influence, public leadership, policy voice or advocacy to shape food environments or seek supportive action from other governments.	Mandates and statutory authority; regulatory limits; planning powers; procurement influence; advocacy to state or federal governments; policy leadership; LG jurisdiction and institutional influence.	General leadership within a partnership; implementation of an existing program without discussion of authority or influence; generic recommendations that government should act.	**P:** LG authority, powers, jurisdiction, influence or advocacy are central.<br>**S:** these are substantive implementation conditions or supporting actions.<br>**N:** absent, generic or inferred only.
9. Consumer behaviour within food environments	Empirical examination or substantive reporting of how people purchase, choose, obtain, prepare, consume or respond to food in relation to environmental conditions, policy or program change.	Purchasing or sales; customer attitudes and acceptability; food-choice constraints; dietary intake linked to environments; coping behaviours; participation as behavioural response; growing and food-acquisition behaviours.	Population dietary statistics as background only; intended behaviour change without measurement; food availability or price alone; community participation without assessment of food behaviour.	**P:** consumer or household behaviour, experience, response or decision-making is central.<br>**S:** behaviour is a supporting outcome or contextual indicator.<br>**N:** behaviour is not substantively examined.

# Boundary rules for commonly overlapping themes: Urban planning vs monitoring - Code monitoring when the document measures or maps spatial access. Add urban planning only when those data are explicitly linked to planning instruments, siting decisions, zoning, development controls, tenure or spatial policy. Intervention vs monitoring - Code intervention when a policy or program is implemented or evaluated. Code monitoring when indicators or audits are collected. Both may apply where monitoring is a substantive part of intervention evaluation. Community engagement vs governance - Code community engagement for participation, lived experience, co-design or community leadership. Code governance for institutional roles, coordination and decision structures. Both may be P when shared governance is genuinely community-led. Governance vs advocacy/powers - Governance concerns how actors coordinate and make decisions. Advocacy/powers concerns authority, jurisdiction, regulatory tools or influence directed within or across government levels. Knowledge translation vs monitoring - Monitoring produces data. Knowledge translation converts data or experience into an accessible tool, profile, guidance or deliberate learning process. A monitoring profile designed for LG action may receive P for both. Food systems/security vs consumer behaviour - Food systems/security concerns structural access and system organisation. Consumer behaviour concerns empirical household or customer choices, purchases, consumption, coping or response. Evidence-based intervention vs policy mapping - A document that only catalogues policies generally receive S or N for interventions. Use P only where intervention implementation, process, acceptability or outcomes are a central focus.

+ P: Primary - The theme is an explicit aim, central analytic focus, principal intervention or policy domain, or a major reported output. Substantive detail is provided on actions, methods, implementation, evaluation, monitoring or findings. Removing the theme would materially change the document's purpose or main conclusions. S: Secondary - The theme is meaningfully discussed and supported by document content but is not a central aim or analytic focus. It may operate as context, an enabling mechanism, a supporting activity, a recommendation, or one component of a broader initiative. More than a passing mention is required. N: Not addressed - No meaningful content maps to the theme, or the theme appears only as a keyword, aspiration, unelaborated reference, background statement or inferred implication. Do not code a theme solely because it is theoretically related to the document topic.

Table S6. Rationale for mapping included studies and reports to local governments' healthy food environment research priorities

Reference (year)	Local governments' research priorities +								
	**1**	**2**	**3**	**4**	**5**	**6**	**7**	**8**	**9**
MacCartan and Palermo (2016)	Not addressed	Not addressed	S: Community participation discussed conceptually, not operationalised	P: LG governance and institutional leadership are the central focus	S: Discusses institutional learning and knowledge use at a high level	Not addressed	S: Food systems referenced as part of public health remit	S: LG authority and constraints discussed, but not advocacy-focus ed	Not addressed
Slade and Carter (2016) 2]	S: Planning acknowledged but not interrogated in depth	S: Policy levers described; no outcome evaluation	S: Participation mentioned, not central	P: Institutional LG role in food policy governance is central	Not addressed	Not addressed	S: Food systems framing present	P: Focus on LG authority and influence	Not addressed
Slade and Carter (2017)	S: Planning tools referenced as part of policy mix	S: Interventions noted, not evaluated	S: Engagement framed conceptually	P: Governance arrangements central	Not addressed	Not addressed	S: Food systems discussed at systems level	P: Explicit focus on statutory and non-statutory powers	Not addressed
Slade and Baldwin (2017)	S: Planning identified as one of several LG levers	S: Interventions referenced but not evaluated	S: Engagement discussed normatively	P: LG leadership and cross-sector governance are central	Not addressed	Not addressed	S: Food systems framed conceptually	P: Explicit focus on LG authority, leadership, and influence	Not addressed
Thornton (2017)	P: Examines spatial planning, land-use, and the built environment as core determinants of food access and equity, with LG planning instruments central to the analysis	Not addressed	S: Community dimensions discussed conceptually	S: Governance appears as context	Not addressed	Not addressed	S: Food access and equity central, but not food security per se	S: Power relations discussed conceptually	S: Consumption patterns discussed at population level
Cuttler et al (2019)	S: Provides spatial affordability data relevant to planning but does not analyse planning tools	S: Generates evidence to inform interventions but does not test one	Not addressed	S: Governance context noted but not analysed	Not addressed	P: Empirical monitoring of food prices and affordability	S: Affordability as one system component	Not addressed	Not addressed

Reeve et al (2020)	S: Planning mechanisms referenced	P: Evaluation of LG food environment interventions	S: Stakeholder engagement acknowledged	P: LG governance structures central	Not addressed	S: Monitoring elements embedded in evaluation	Not addressed	S: Policy authority discussed implicitly	Not addressed
Riesenberg et al (2020)	Not addressed	P: Retail policy intervention central	Not addressed	P: LG policy governance central	Not addressed	P: Uses policy surveys and audits	Not addressed	S: LG authority implicit	S: Consumer acceptability examined indirectly
Blake et al (2021)	Not addressed	P: Core focus on intervention effectiveness	Not addressed	Not addressed	Not addressed	S: Monitoring as part of evaluation	Not addressed	Not addressed	P: Customer attitudes and responses assessed
Carrad et al (2022)	P: Systematically analyses LG planning and land-use policies	S: Identifies policy instruments that enable interventions but does not evaluate effectiveness	S: Acknowledge engagement and participation, but neither empirically studies co-design processes	P: Governance arrangements and cross-sector roles of LG are central	Not addressed	P: Explicitly monitors and categorises LG food-related policies across jurisdictions	S: Situate LG food policies within broader food system and food security objectives, without being food-security–specific studies	S: Explicitly analyses LG authority, limits, and advocacy potential	Not addressed
Carrad et al (2022)	S: Discusses planning as an enabling lever rather than a core object of analysis	Not addressed	S: Acknowledge engagement and participation, but neither empirically studies co-design processes	P: Governance arrangements and cross-sector roles of LG are central	S: Touches on the need for clearer role articulation and learning across government	Not addressed	S: Situate LG food policies within broader food system and food security objectives, without being food-security–specific studies	P: explicitly analyses LG authority, limits, and advocacy potential	Not addressed
Love et al (2022)	S: Discusses planning levers conceptually within food systems policy	S: Identifies intervention opportunities without outcome evaluation	Not addressed	P: Strong focus on governance arrangements and coordination	Not addressed	Not addressed	P: Explicit food systems framing	S: Notes role of influence	Not addressed
Riesenberg et al (2022)	Not addressed	S: Describes policy presence, not intervention effectiveness	Not addressed	S: Describes institutional roles and responsibilities	Not addressed	S: Survey data on policy presence, not environments	Not addressed	S: Policy authority discussed descriptively	Not addressed
Barbour et al (2023)	S: Planning context discussed as an enabler in a governance case study	Not addressed	Not addressed	P: CFIR-based analysis of multisectoral governance	Not addressed	Not addressed	S: Food systems framing via strategy	S: Leadership and influence noted	Not addressed

Carrad et al (2023)	P: Explicitly analyses LG use of planning, zoning, land-use and regulatory levers	S: Catalogues interventions but does not assess effectiveness	S: Mentions engagement practices but does not focus on co-design	P: Central focus on governance systems and partnerships	Not addressed	S: Identifies lack of monitoring	P: Whole-of-system LG analysis	P: Explicit discussion of LG mandates and advocacy	Not addressed
Dangerfield et al (2023)	S: Reviews MPHWPs which include planning actions, but does not evaluate planning tools	Not addressed	Not addressed	S: Governance reflected indirectly via MPHWPs	Not addressed	S: Documents absence of indicators in plans	S: Public health plans include food access	Not addressed	Not addressed
Day et al (2023)	Not addressed	S: Interventions discussed via workforce role	S: Co-design potential discussed but constrained	S: Governance appears as system barrier	S: Identifies lack of decision-support tools	Not addressed	S: Food systems context acknowledged	Not addressed	Not addressed
Molenaar et al (2023)	S: Community food access issues linked to place, not planning mechanisms	S: Generates evidence to inform interventions, does not implement them	P: Photovoice explicitly centres lived experience and co-produced knowledge	S: LG governance acknowledged but not analysed	Not addressed	S: Uses food security measurement, not environment monitoring	P: Core focus on food insecurity and access	S: Policy implications noted	P: Rich empirical evidence on food choice, coping, constraints
Blake et al (2025)	Not addressed	P: Core focus on intervention effectiveness	Not addressed	Not addressed	Not addressed	S: Monitoring as part of evaluation	Not addressed	Not addressed	P: Customer attitudes and responses assessed
Godrich et al (2025)	Not addressed	S: Describes initiatives but explicitly notes effectiveness not assessed	P: Place-based, community-embedded partnerships are central	P: Partnerships are the core analytic focus	Not addressed	Not addressed	P: Food security systems central	S: LG influence acknowledged but not analysed	Not addressed
Godrich et al (2025)	Not addressed	Not addressed	P: Co-creation and two-way government–community engagement are central analytic foci	P: Examines governance arrangements and government–community interfaces as drivers of systems change	P: Knowledge sharing, sense-making, and collective learning are explicit aims of the systems-change approach	Not addressed	P: Food security and food systems change are the core objects of analysis	S: Advocacy pathways are discussed as gaps rather than analysed as primary outcomes	Not addressed
Keegan et al (2025)	S: Planning appears as one of several policy levers supporting resilience	Not addressed	S: Collaborative governance is discussed conceptually, not empirically studied	P: Multi-level governance and policy integration are central to the analysis	S: Social and policy learning are discussed as mechanisms	Not addressed	P: Food system resilience and security are the main analytical focus	S: LG authority is discussed in relation to limitations and constraints	Not addressed

Pickles et al (2025)	P: Land access, zoning, and planning constraints are central findings	Not addressed	S: Strong grassroots participation, but no formal LG co-design processes	S: LG role discussed as enabling context rather than central focus	Not addressed	Not addressed	P: Civic food networks and community gardening are analysed as contributors to local food security and resilience	Not addressed	Not addressed
Heart Foundation Tasmania (2016)	S: Planning and spatial access (shop location, transport, walkability) discussed descriptively, not as planning instruments	Not addressed	S: Community gardens, markets and programs described; no formal co-design processes analysed	S: Identifies roles of councils, NGOs, schools and services, but governance is not the analytical focus	P: Purpose is to translate data into accessible profiles to inform LG and community action	P: Systematic monitoring of food access, affordability, outlets and basket costs at LGA level	P: Food security, access, affordability and resilience are central aims	S: Implicit advocacy role suggested; statutory powers not analysed	S: Consumption patterns (fruit/vegetable intake) reported descriptively
Heart Foundation Tasmania (2016)	S: Planning and spatial access (shop location, transport, walkability) discussed descriptively, not as planning instruments	Not addressed	S: Community gardens, markets and programs described; no formal co-design processes analysed	S: Identifies roles of councils, NGOs, schools and services, but governance is not the analytical focus	P: Purpose is to translate data into accessible profiles to inform LG and community action	P: Systematic monitoring of food access, affordability, outlets and basket costs at LGA level	P: Food security, access, affordability and resilience are central aims	S: Implicit advocacy role suggested; statutory powers not analysed	S: Consumption patterns (fruit/vegetable intake) reported descriptively
Heart Foundation Tasmania (2016)	S: Planning and spatial access (shop location, transport, walkability) discussed descriptively, not as planning instruments	Not addressed	S: Community gardens, markets and programs described; no formal co-design processes analysed	S: Identifies roles of councils, NGOs, schools and services, but governance is not the analytical focus	P: Purpose is to translate data into accessible profiles to inform LG and community action	P: Systematic monitoring of food access, affordability, outlets and basket costs at LGA level	P: Food security, access, affordability and resilience are central aims	S: Implicit advocacy role suggested; statutory powers not analysed	S: Consumption patterns (fruit/vegetable intake) reported descriptively
Heart Foundation Tasmania (2016)	S: Planning and spatial access (shop location, transport, walkability) discussed descriptively, not as planning instruments	Not addressed	S: Community gardens, markets and programs described; no formal co-design processes analysed	S: Identifies roles of councils, NGOs, schools and services, but governance is not the analytical focus	P: Purpose is to translate data into accessible profiles to inform LG and community action	P: Systematic monitoring of food access, affordability, outlets and basket costs at LGA level	P: Food security, access, affordability and resilience are central aims	S: Implicit advocacy role suggested; statutory powers not analysed	S: Consumption patterns (fruit/vegetable intake) reported descriptively

Heart Foundation Tasmania (2016)	S: Planning and spatial access (shop location, transport, walkability) discussed descriptively, not as planning instruments	Not addressed	S: Community gardens, markets and programs described; no formal co-design processes analysed	S: Identifies roles of councils, NGOs, schools and services, but governance is not the analytical focus	P: Purpose is to translate data into accessible profiles to inform LG and community action	P: Systematic monitoring of food access, affordability, outlets and basket costs at LGA level	P: Food security, access, affordability and resilience are central aims	S: Implicit advocacy role suggested; statutory powers not analysed	S: Consumption patterns (fruit/vegetable intake) reported descriptively
Heart Foundation Tasmania (2016)	S: Planning and spatial access (shop location, transport, walkability) discussed descriptively, not as planning instruments	Not addressed	S: Community gardens, markets and programs described; no formal co-design processes analysed	S: Identifies roles of councils, NGOs, schools and services, but governance is not the analytical focus	P: Purpose is to translate data into accessible profiles to inform LG and community action	P: Systematic monitoring of food access, affordability, outlets and basket costs at LGA level	P: Food security, access, affordability and resilience are central aims	S: Implicit advocacy role suggested; statutory powers not analysed	S: Consumption patterns (fruit/vegetable intake) reported descriptively
Heart Foundation Tasmania (2016)	S: Planning and spatial access (shop location, transport, walkability) discussed descriptively, not as planning instruments	Not addressed	S: Community gardens, markets and programs described; no formal co-design processes analysed	S: Identifies roles of councils, NGOs, schools and services, but governance is not the analytical focus	P: Purpose is to translate data into accessible profiles to inform LG and community action	P: Systematic monitoring of food access, affordability, outlets and basket costs at LGA level	P: Food security, access, affordability and resilience are central aims	S: Implicit advocacy role suggested; statutory powers not analysed	S: Consumption patterns (fruit/vegetable intake) reported descriptively
Heart Foundation Tasmania (2016)	S: Planning and spatial access (shop location, transport, walkability) discussed descriptively, not as planning instruments	Not addressed	S: Community gardens, markets and programs described; no formal co-design processes analysed	S: Identifies roles of councils, NGOs, schools and services, but governance is not the analytical focus	P: Purpose is to translate data into accessible profiles to inform LG and community action	P: Systematic monitoring of food access, affordability, outlets and basket costs at LGA level	P: Food security, access, affordability and resilience are central aims	S: Implicit advocacy role suggested; statutory powers not analysed	S: Consumption patterns (fruit/vegetable intake) reported descriptively
Tasmanian Department of Health and Human Services (2016)	S: LG planning roles discussed in findings, not analysed	S: Synthesises practitioner evidence on "what works"	P: Built entirely on stakeholder and community consultation	P: Governance, whole-of-government and partnership effectiveness analysed	S: Findings intended to inform policy	S: Monitoring discussed at system level	P: State-wide food security systems analysis	P: LG advocacy roles and limits analysed	S: Behavioural change discussed via stakeholder accounts

North East Local Food Strategy Working Group (2019)	P: Planning reform, land-use, logistics and procurement are central strategy pillars	S: Strategic directions informed by evidence, no intervention testing	S: Extensive consultation informed strategy, but governance focus dominates	P: Cross-sector governance and regional coordination are foundational	P: Strategic framework to guide action and funding	S: Uses secondary data; not a monitoring system	P: Comprehensive food-systems strategy	P: Advocacy and LG powers are a core theme	S: Behaviour discussed in consumption domain
Latrobe Health Assembly (2021)	S: Planning referenced through coalition actions, not central	S: Describes interventions but does not assess effectiveness	P: Community-led collective impact model and co-design processes central	P: Governance and coalition functioning are the core analytic focus	S: Reporting and accountability rather than guidance	P: Ongoing monitoring of food security indicators reported	P: Food security systems focus	S: Advocacy role noted but not analysed	S: Behaviour inferred through participation
The Sax Institure (2021)	Not addressed	Not addressed	P: Community-led collective impact model and co-design processes central	P: Aboriginal-led governance and partnership principles central	P: Designed as a planning and discussion tool	Not addressed	P: Systems approach to Aboriginal food security	S: Advocacy encouraged via leadership	S: Food practices discussed qualitatively
Bendigo Health - Loddon Mallee Public Health Unit (2024)	Not addressed	S: Capacity-building intervention described; outcomes are self-reported	P: Capacity-building workshops and community mapping are core	S: Reference group and partnerships described	S: Knowledge sharing and learning focus	P: Survey-based food system monitoring	P: Local food systems and food security focus	Not addressed	P: Survey data directly capture purchasing, growing and access behaviours
Food Connect Foundation (2025)	S: Land, infrastructure siting and zoning implications discussed conceptually	S: Presents case examples and models; not a controlled evaluation	P: Community ownership, participation and relational governance are core to the model	P: Multi-level governance, cross-sector partnerships and Indigenous governance are central	P: Explicit aim to codify, share and replicate practice-based knowledge nationally	**Not addressed**	P: Central focus on resilient, regenerative food systems and infrastructure	P: Strong emphasis on procurement, policy influence and the role of government (including LG)	S: Consumer access and values referenced, not analysed behaviourally
Brimbank City Council (2025)	S: Built environment considered as a determinant, not via planning tools	S: Strategic responses outlined, not intervention evaluations	S: Consultation and partnership acknowledged; not co-design-focused	P: Statutory LG role, policy alignment and partnerships are explicit and central	P: Designed to synthesise evidence to guide MPHWP priorities and action	P: Robust monitoring of food insecurity, diet, retail exposure and related determinants	P: Food insecurity and healthy eating are identified as priority action areas	S: Advocacy role acknowledged (legislative and policy alignment), not analysed in depth	S: Dietary behaviours reported at population level

Considerations: i) Not addressed: No meaningful content that maps to the stated priority theme ii) Secondary relevance: The document includes some content relevant to the stated priority theme, but the theme is not the main focus (eg, mentioned briefly or embedded within a broader strategy such as general wellbeing/sustainability) iii) Primary relevance: The document addresses the stated priority theme (explicitly or clearly as the central focus) and includes substantive detail (eg, actions, implementation, evaluation, monitoring)

+ Local governments' research priorities: 1. Urban planning strategies, tools and guidelines; 2. Evidence-based interventions for healthy food environments; 3. Community engagement and co-design; 4. Governance and multisectoral partnerships; 5. Knowledge translation and dissemination; 6. Food environment monitoring; 7. Food systems and food security; 8. Advocacy and local government powers; 9. Consumer behaviour within food environments.
